# Development of a Novel Immune-Related Gene Prognostic Index for Breast Cancer

**DOI:** 10.3389/fimmu.2022.845093

**Published:** 2022-04-26

**Authors:** Yan Yao, Xinru Kong, Ruijuan Liu, Fei Xu, Gongxi Liu, Changgang Sun

**Affiliations:** ^1^ College of First Clinical Medicine, Shandong University of Traditional Chinese Medicine, Jinan, China; ^2^ Innovative Institute of Chinese Medicine, Shandong University of Traditional Chinese Medicine, Jinan, China; ^3^ Department of Oncology, Weifang Traditional Chinese Hospital, Weifang, China; ^4^ Department of Geriatric Medicine, Affiliated Hospital of Shandong University of Traditional Chinese Medicine, Jinan, China; ^5^ College of Traditional Chinese Medicine, Weifang Medical University, Weifang, China

**Keywords:** breast cancer, immune-related genes, prognostic index, WGCNA, immune checkpoint inhibitor

## Abstract

**Objective:**

To construct an immune-related gene prognostic index (IRGPI) for breast cancer (BC) and investigate its prognostic specificity and the molecular and immune characteristics.

**Methods:**

BC hub genes were identified from The Cancer Genome Atlas and immune-related databases using weighted gene co-expression network analysis (WGCNA). IRGPI was constructed using univariate, LASSO, and multivariate regression analyses, and was validated with GSE58812 and GSE97342 in the Gene Expression Omnibus database (GEO). At the same time, we evaluated the predictive ability of IRGPI for different BC subtypes. Subsequently, the molecular and immune characteristics, clinical relevance, and benefits of immune checkpoint inhibitor treatment were analyzed for different IRGPI subgroups.

**Results:**

IRGPI consisted of six genes: SOCS3, TCF7L2, TSLP NPR3, ANO6, and HMGB3. The IRGPI 1-, 5-, and 10-years area under curve (AUC) values were 0.635, 0.752, and 0.753, respectively, indicating that IRGPI has good potential in predicting the long-term survival of BC patients, consistent with the results in the GEO cohort. IRGPI showed good predictive power in four different breast cancer subtypes: ER positive, PR positive, HER2 positive and triple-negative (*P*<0.01). Compared with the low-IRGPI group, the high-IRGPI group had a worse prognosis and a lower degree of immune infiltrating cells (*p* < 0.05). IRGPI showed specificity in distinguishing age, TNM stage, ER, and HER2 statuses, and our study found that the high-IRGPI group had low tumor immune dysfunction and exclusion (TIDE), microsatellite instability (MSI), and T cell dysfunction scores (*p* < 0.05). In addition, compared with the TIDE and TIS models, showed that the AUCs of IRGPI were better during the 5-year follow-up.

**Conclusion:**

IRGPI can be used as an independent prognostic indicator of breast cancer, providing a method for monitoring the long-term treatment of BC.

## Introduction

Breast cancer (BC) is the most common malignant tumor in women. According to the latest statistics, the number of new cases of BC in the world is 2.26 million, surpassing the 2.2 million cases of lung cancer, thereby becoming the most common cancer worldwide (1). BC is the leading cause of cancer-related deaths among women, accounting for 30% of newly diagnosed cases and 15% of cancer-related deaths (2). Traditional treatments for BC, such as surgery, radiotherapy, chemotherapy, and endocrine therapy, often cause adverse side effects that are difficult for patients to tolerate (3–5). In recent years, immunotherapy has achieved great success in the treatment of melanoma, lung cancer, acute lymphoblastic leukemia, and other tumors (6). Although BC was previously considered as a poor immunogenic cancer, patients with BC are now expected to benefit from immunotherapies.

Immunotherapy involving PD-1/PD-L1 inhibitor treatment has shown promising results in patients with advanced BC and triple-negative BC (7). Moreover, anti-PD-1/PD-L1 antibody, anti-CTLA-4 antibody, anti-LAG-3 antibody, and other immune checkpoint inhibitor can inhibit tumor escape, thereby providing a new strategy for the treatment of BC (8–10). Therefore, the identification of potential prognostic markers associated with therapeutic benefits can help personalize immunotherapy in BC patients. Although there are several studies on the application of immunotherapies in BC, only a small number of patients have responded to treatments, and the underlying mechanism of immunotherapy in BC remains poorly understood (11). In addition, effective clinical biomarkers for BC are currently lacking. Therefore, there is an unmet need for a comprehensive understanding of the interactions between BC and the immune system, which can help identify potential immuno-oncological prognostic and predictive markers of BC.

New biomarker combinations are the basis for increasingly complex diagnostic algorithms. In addition, multigene prognostic models can guide physicians in choosing appropriate treatments. In this study, we constructed a novel immune-related gene prognostic index (IRGPI) for BC. The highly correlated modules of breast cancer were selected by integrating the transcriptome analysis of immune-related genes with weighted gene co-expression network analysis (WGCNA). We established a prognostic model of immune-related genes using gene enrichment analysis and Cox regression analysis. Moreover, we further verified the reliability of our model, described the molecular and immune characteristics of different IRGPI subsets, carried out a correlation analysis between clinical symptoms and immune subtypes, and compared our model with other models. Overall, our study showed that IRGPI is a promising prognostic biomarker.

## Materials and Methods

### Clinical Samples and Data Collection

Individual BC transcriptome RNA-sequencing (RNA-seq) data (Htseq-FPKM) and corresponding clinical data were obtained from The Cancer Genome Atlas (TCGA) database. The data collection date was November 9, 2021. The list of immune-related genes (IRGs) was acquired from the ImmPort (https://www.immport.org/shared/hom) and InnateDB (https://www.innatedb.com/) databases. Validation group data was from the Gene Expression Omnibus database (GEO) (GSE58812 and GSE97342).

### BC Immune-Related Hub Gene Acquisition

The acquisition of BC hub IRGs involved the following: 1) Collating and standardizing RNA transcriptome sequencing data. The Limma package in R software was used to analyze significantly differentially expressed genes (DEGs), with | log2FoldChange| (FC) > 1 and a false discovery rate <0.05 as the cut-off values. 2). All IRGs in ImmPort and InnateDB database were combined, the online site Venny 2.1.0 [Venny 2.1.0 (csic.es)] was used to extract differentially expressed IRGs from the intersection of immune genes and all DEGs. 3) WGCNA was used to identify highly covarying gene sets and candidate biomarker genes or therapeutic targets were identified based on the interconnectedness of the gene sets and the association between gene sets and phenotypes. First, a cluster tree was drawn, abnormal samples were removed, the Pearson correlation coefficient between the two genes was calculated, and a similarity matrix was constructed using expression data. Then, a power of β value was introduced to transform the similarity matrix into an adjacency matrix. The power of the β value was six. Based on this, we constructed a scale-free network and topological overlap matrix (TOM), which is used to describe the correlation degree between genes. Subsequently, a hierarchical clustering tree of genes (dendrogram) of the hclust function was generated through hierarchical clustering for module detection. Finally, the dynamic tree cutting method was used to generate the final module, and the main parameters were: deepSplit = 2, minModuleSize = 20, threshold = 0.3, and MEDissThres = 0.25. The genes in the module with the highest correlation were selected as hub genes for further analysis.

### Functional Enrichment Analysis and Protein- Protein Interaction (PPI) Network Analysis

For the highly correlated module genes obtained from WGCNA analysis, the clusterProfiler package was used to conduct gene ontology (GO) enrichment and Kyoto Encyclopedia of Gene and Genome (KEGG) pathway analysis. The results of GO analysis mainly consisted of three parts: biological processes (BP), cellular components (CC), and molecular functions (MF). *P* < 0.05 was the screening criterion, and the top 30 items with the lowest *p-*value were selected and visualized as bubble charts by the “ggplot2” package in R. Module genes were simultaneously used for PPI network analysis. The STRING online network database was used to query the relationship between proteins, with a cut-off criterion confidence score ≥0.90, and the results were visualized using Cytoscape software version 3.8.0.

### Constructing and Validating the IRGPI

The IRGPI was developed as follows: 1) Survival time and survival status in BC clinical data was integrated with gene expression levels of key genes in the module. Univariate Cox proportional hazard regression analysis was performed for key genes using the survival package of R, and genes with *p* < 0.05. 2) To achieve variable selection and dimension reduction, the “glmnet” package of R was used for LASSO Cox regression analysis. In this process, a random 1000 cross-validation routine was used to select the penalty regularization parameter λ, and lambda.min was the lambda value that produced the minimum mean cross-validated error. 3) The results of the LASSO algorithm were used for multivariate Cox proportional hazards regression analysis. Based on the results of the multivariate Cox analysis, a prognostic index (PI) was constructed to evaluate the prognostic risk of BC patients. The risk score (RS) of each patient was calculated according to the PI, RS = Σexpgenei* βi, and the corresponding median risk score was used as the boundary value to divide the sample into high-risk and low-risk groups. Kaplan-Meier (K-M) survival curve, log-rank test, and univariate and multivariate analyses were used to evaluate the independent prognostic ability of the IRGPI, at the same time, the GEO cohort as verification group to verify the reliability of the results. 4) Based on the clinical information of BC samples, ER positive, PR positive, HER2 positive and triple-negative different BC subtypes were extracted respectively. The prediction ability of IRGPI in different breast cancer subtypes was further evaluated. In addition, using the survival ROC package in R, we constructed 1-, 5- and 10-year time-dependent receiver operating characteristic (ROC) curves to estimate the area under the curve (AUC) for the PI. Meanwhile, we compared the validation of IRGPI *vs.* PD-L1 as a predictor for survival.

### Comprehensive Analysis of Molecular and Immune Characteristics in Different IRGPI Subgroups

Gene set enrichment analysis (GSEA) was performed using the Limma package of R to further explore the molecular mechanisms underlying the prognostic difference between high- and low-risk groups. GO and KEGG gene sets were obtained from the GSEA online database (Gsea-msigdb.org). Statistical significance was set at *p*-value < 0.05.

The CIBERSORT algorithm (http://cibersort.stanford.edu/) was used to assess the relative proportions of 22 types of invasive immune cells in the BC-standardized gene expression data. Differential analysis of immune cells in the high- and low-risk groups was performed using R’s Limma package. To explore the relationship between immune cells and survival, all samples were divided into high and low expression groups according to the content of immune cells, and the survival package and survminer package were used to compare the survival differences between the groups. We used the Limma, GAVA, and GSEABase packageS of R to analyze the differences in immune-related functions and survival for different IRGPI subgroups. The screening criterion was set as *p* < 0.05.

### Correlation Analysis of IRGPI Grouping With Clinical Symptoms and Immune Subtypes

To evaluate the clinical value of IRGPI, we integrated the clinical data of BC, used R’s RcolorBrewer package and chi-square test to observe whether there were differences in clinical traits between high- and low-risk groups, including age, pathological stage, T stage, N stage, M stage, ER, PR, and HER2 status. We also evaluated the relationship between the immune subtypes of BC and IRGPI in the TCGA database. Meanwhile, the limma package of R was used to analyze the difference and correlation between IRGPI and PD-L1 expression.

### Immunotherapy in IRGPI Subgroups

Tumor immune dysfunction and exclusion (TIDE) can be used to identify biomarkers to predict the efficacy of immune checkpoint inhibitors (ICIs) by comprehensive analysis of tumor expression profiles. A higher TIDE prediction score represented a higher potential for immune evasion, which suggested that the patients were less likely to benefit from ICI therapy. We used TIDE (http://tide.Dfci.harvard.edu/) to evaluate the potential clinical efficacy of immunotherapy in different IRGPI subgroups. TIDE, microsatellite instability (MSI), and T cell exclusion and dysfunction scores were compared using the Wilcoxon test. In addition, we performed a time-dependent ROC curve analysis using R’s timeROC package to obtain AUC and compared the prognostic value between TIDE and TIS with IRGPI.

## Results

### Identification of Differentially Expressed IRGs

We downloaded the transcriptome data of 1,109 BC samples and 113 normal samples from the TCGA database and performed a differential analysis using the R’s Limma package. We identified a total of 5,456 DEGs, including 3,195 upregulated and 2,261 downregulated DEGs (| log2FC | > 1 and *p* < 0.05). The results are shown in **Supplementary Figure 1A**. GSE58812 and GSE97342 include 135 BC samples. A list of BC IRGs was acquired using the ImmPort and InnateDB online databases, and 530 differentially expressed IRGs were identified, including 260 downregulated and 270 upregulated IRGs (**Supplementary Figure 1B**).

### Identification of Immune-Related Hub Genes

**Figure 1:**
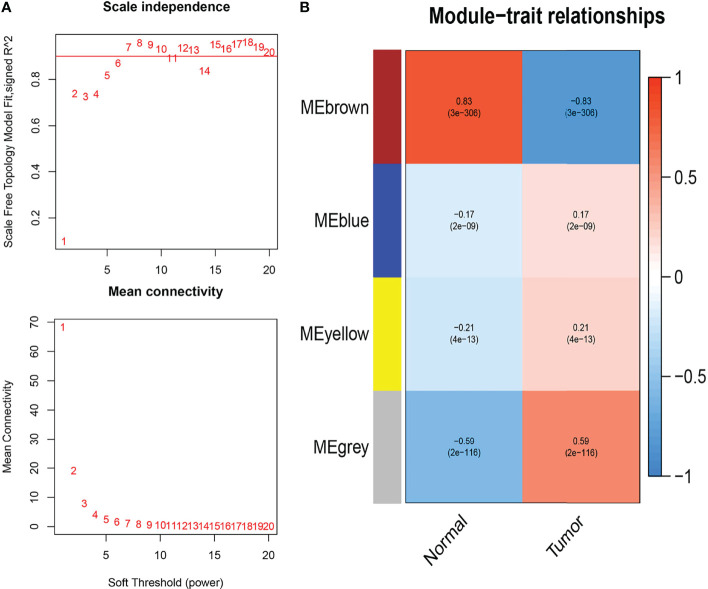
Weighted co-expression network analysis (WGCNA) of immune-related differential genes. **(A)** Determining the soft threshold power in WGCNA analysis. **(B)** Gene modules related to BC obtained by WGCNA.

We acquired the expression profiles of the 530 differentially expressed IRGs and constructed the co-expression network using the WGCNA package in R software. The optimal soft-thresholding power was six based on a scale-free network (**Figure 1A**). In the network, the minimum number of modules was set at 20, the parameter of MEDissThres was set at 0.25, and the threshold was set at 0.3. We calculated and plotted the relationship between each module and its corresponding clinical features. Finally, four modules were obtained; the brown modules shown in **Figure 1B** showed the strongest negative correlation with tumor samples associated with the IRG co-expression network (module-feature weighted correlation = 83). A total of 196 genes in the brown module were selected as hub genes for further analysis.

### PPI Network Construction and Gene Enrichment Analysis

To further explore the interaction between the 196 hub genes, we performed a PPI network analysis. First, we preprocessed the genes using the STRING online database, set the cut-off standard confidence score at ≥0.90, and removed discrete genes. We then visualized the results, which included 88 nodes and 320 edges, using Cytoscape software (**Supplementary Figure 2A**). When a specific gene has more connections than others, it is considered to have important biological functions. Therefore, we used the Barplot package of R to visualize 15 genes with the most connections in the network (**Supplementary Figure 3B**). The nodes with the highest connectivity in the network were PIK3R1, FOS, and JUN.

**Figure 2:**
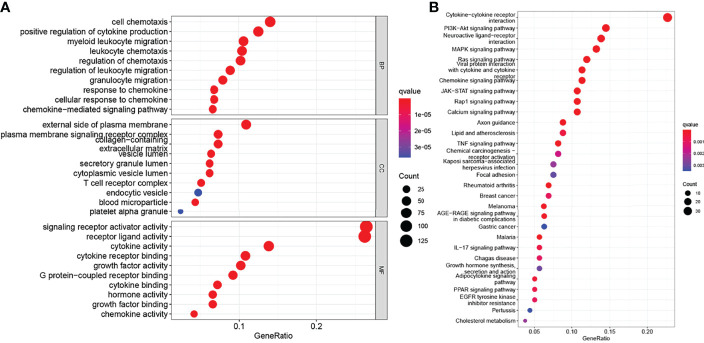
Functional enrichment of genes in the brown module. **(A)** Gene Ontology (GO) functional enrichment, include biological process (BP), cellular component (CC), and molecular function (MF). **(B)** Kyoto Encyclopedia of Genes and Genomes (KEGG) pathways enrichment analysis (*P* < 0.05).

In addition, to explore the function of these hub genes, we used the clusterProfiler package in R for gene enrichment analysis, and obtained a total of 2284 GO items (**Supplementary Table 1**), including 2110 BP, 37 CC, and 137 MF terms and 104 KEGG pathway items (**Supplementary Table 2**). The 30 items with the lowest *p*-value were selected and visualized as bubble graphs by R’s “GGplot2” package. GO analysis showed that these genes were mainly involved in biological processes such as chemotaxis, migration, proliferation, and apoptosis; cellular components such as extracellular matrices, plasma membranes, and proteins; and biological functions such as receptor, ligand activity, and cytokine activities (**Figure 2A**). KEGG pathway analysis showed that these genes were involved in signaling pathways, including cytokine-cytokine receptor interaction, JAK-STAT, chemokine signaling pathways (**Figure 2B**).

### Development of IRGPI

**Figure 3:**
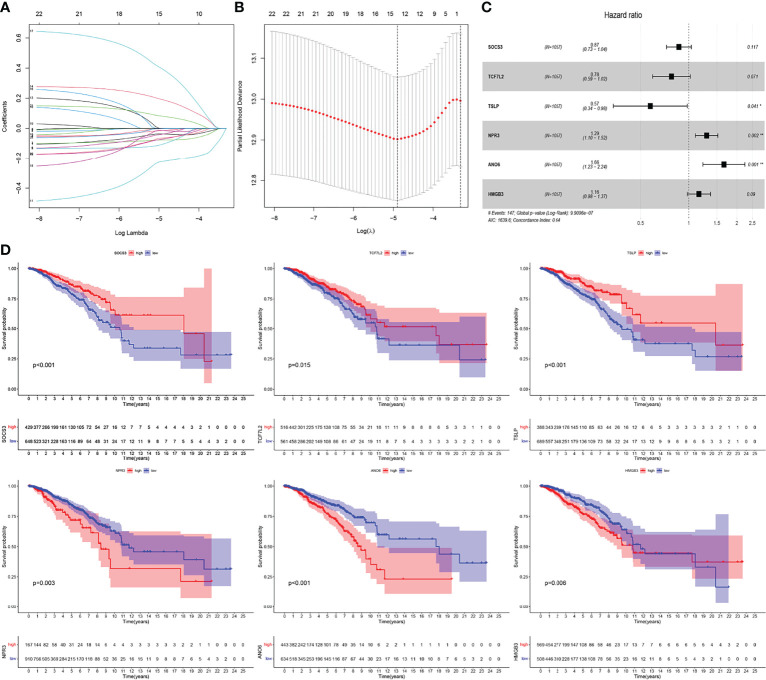
Construction of immune-related prognostic index (IRGPI) for BC. **(A)** LASSO coefficient profiles of the IRGs associated with disease-free survival of BC. **(B)** Plots of the cross-validation error rates. Each dot represents a lambda value along with error bars that represent the confidence interval for the cross-validated error rate. **(C)** Forest plots of hazard ratios (HR) of survival-associated IRGs obtained using multivariate Cox regression analysis. **(D)** K-M survival curves of 6 genes. *P <0.05, **P <0.01

To develop an IRGPI, we performed the following: 1) We first applied the univariate Cox proportional risk regression analysis on 196 IRGs and acquired 23 genes (*p* < 0.05) (**Table 1**). 2) We performed the LASSO Cox regression analysis on 23 genes with a 1000-fold cross-validation (**Figure 3A**) and identified 14 IRGs (**Figure 3B**). 3) We then performed multivariate Cox proportional risk regression analysis on these 14 IRGs and identified six IRGs: three protective factors SOCS3, TCF7L2, and TSLP; and three risk factorsNPR3, ANO6, and HMGB3 (**Figure 3C**). Moreover, we simultaneously performed the K-M survival analysis on the six IRGs. Our results showed that the *p-*values of the six IRGs were less than 0.05; low expression of SOCS3, TCF7L2, and TSLP, and high expression of HMGB3 in BC was associated with poor prognosis; and low expression of NPR3 and ANO6 in BC was associated with good prognosis (**Figure 3D**). Using Kaplan-Meier Plotter (http://kmplot.com/analysis/index.php?p=service), CSCA (http://bioinfo.life.hust.edu.cn/GSCA/#/expression), GEPIA2 (http://gepia2.cancer-pku.cn/#survival) online database conducted an overall survival analysis of 6 genes, which verified our results (P<0.05). Further, we calculated the RS and constructed the IRGPI of BC based on the expression level and regression coefficient of the six survival-related IRGs.

**Table 1:** Results of univariate Cox proportional risk regression analysis of 196 IRGs (P<0.05).

Genes	HR	HR.95L	HR.95H	pvalue
TSLP	0.745	0.292	0.775	0.003
S100B	0.841	0.748	0.946	0.004
SOCS3	0.791	0.674	0.929	0.004
TP63	0.786	0.661	0.935	0.006
ANO6	1.477	1.105	1.972	0.008
FREM1	0.606	0.407	0.903	0.014
NPR3	1.212	1.039	1.415	0.015
IL33	0.85	0.745	0.969	0.015
TACR1	0.71	0.539	0.936	0.015
JUN	0.823	0.698	0.971	0.021
STAT5A	0.785	0.635	0.969	0.024
SERPING1	0.828	0.702	0.977	0.025
NGFR	0.852	0.74	0.981	0.026
HMGB3	1.187	1.019	1.382	0.027
LIFR	0.809	0.669	0.978	0.028
RBP4	0.884	0.79	0.988	0.03
VIM	0.811	0.671	0.98	0.031
RGS2	0.869	0.764	0.987	0.031
CCL23	0.652	0.44	0.966	0.033
ICAM2	0.765	0.594	0.986	0.038
C3	0.888	0.792	0.997	0.044
SEMA3G	0.85	0.724	0.998	0.048
TCF7L2	0.794	0.631	0.999	0.049

**Figure 4:**
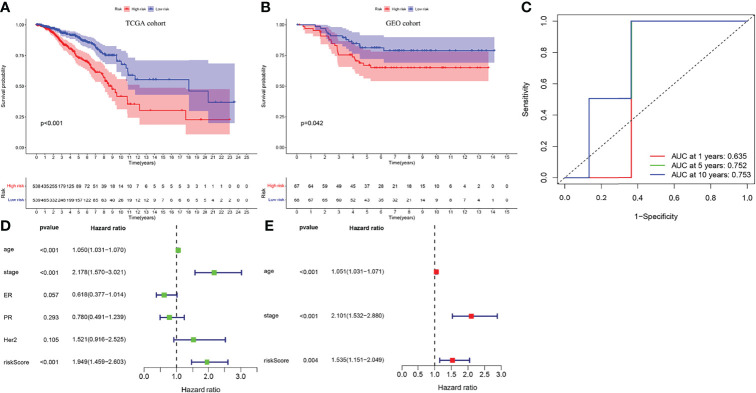
The verification of IRGPI. **(A)** Survival curves of BC patients with high-IRGPI and low-IRGPI subgroups in TCGA cohort. **(B)** Survival curves of BC patients with high-IRGPI and low-IRGPI subgroups in GEO cohort. **(C)** The IRGPI 1, 5and 10 years area under curve (AUC). **(D)** IRGPI univariate COX regression analysis of independent prognosis. **(E)** IRGPI multivariate COX regression analysis of independent prognosis.

All BC samples were divided into high- and low-risk groups based on the median RS. The survival analysis showed that the overall survival (OS) of high-risk patients was significantly lower than that of low-risk patients (*p* < 0.01) (**Figure 4A**), consistent with the results in the GEO cohort (*p* = 0.042) (**Figure 4B**). IRGPI showed good predictive power in four different breast cancer subtypes: ER positive, PR positive, HER2 positive and triple-negative (P<0.01) (**Supplementary Figures 3A-D**). To verify the accuracy of IRGPI prediction, the receiver operating characteristics (ROC) curves for 1, 5, and 10 years were drawn, and the AUC was calculated. The AUC of IRGPI for 1, 5, and 10 years was 0.635, 0.752, and 0.753, respectively (**Figure 4C**), indicating that IRGPI has good potential in predicting long-term survival of BC patients. while the AUC of PD-L1 at 1, 5 and 10 years was 0.612, 0.542 and 0.555, respectively (**Supplementary Figure 3E**). Our results showed that IRGPI had better predictive ability than PD-L1 expression. In addition, we verified IRGPI by univariate (**Figure 4D**) and multivariate (**Figure 4E**) Cox regression analyses combined with clinical factors, which showed that IRGPI had statistical differences and could be used as an independent prognostic factor.

### Molecular and Immune Characteristics of Different IRGPI Subgroups

**Figure 5:**
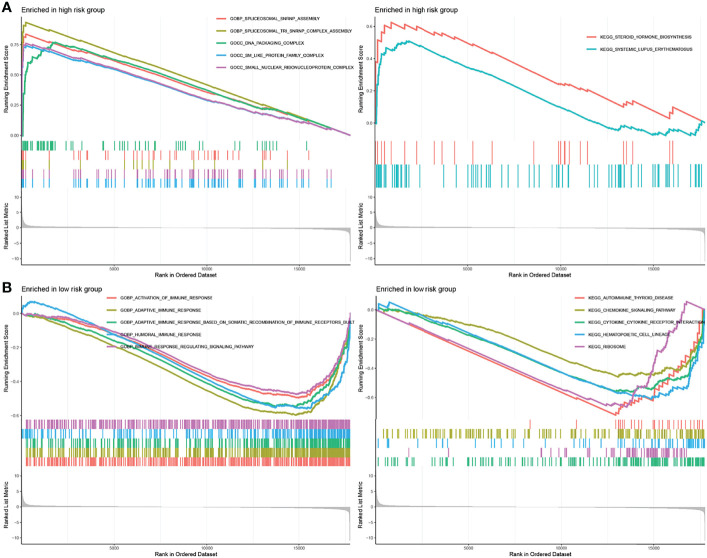
GSEA analysis of different IRGPI subgroups. **(A)** GSEA enriched in high-IRGPI subgroup. **(B)** GSEA enriched in low-IRGPI subgroup. (*P* < 0.05).

To identify gene sets enriched in different IRGPI subgroups, we used GSEA. Our results showed that genes in the high-IRGPI group were mainly enriched in spliceosomal assembly and protein complex (**Figure 5A**), whereas those in the low-IRGPI group were enriched in immune response and the regulation of related signaling pathways (**Figure 5B**).

**Figure 6:**
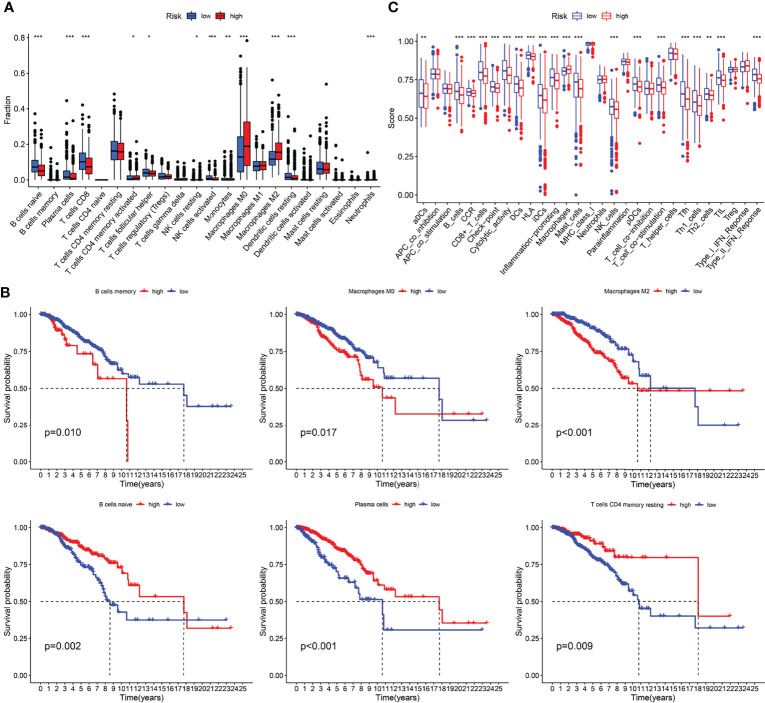
Immune characteristics analysis of different IRGPI subgroups. **(A)** The proportions of immune infiltration cells in different IRGPI subgroups. **(B)** Kaplan-meier survival curve of differential immune cells (*P* < 0.05). **(C)** The difference immune cell function in different IRGPI subgroups. *P <0.05, **P <0.01, ***P <0.001.

Furthermore, to analyze the composition and function of immune cells in different IRGPI subgroups, we used the CIBERSORT algorithm to evaluate the differences in 22 types of invasive immune cells in different IRGPI subgroups. We found that M0 and M2 macrophages were more abundant in the high IRGPI subgroup, whereas in low-IRGPI subgroups, naive B cells, plasma cells, and CD8 T cells were more abundant (**Figure 6A**). We then conducted survival analysis of the different immune cells and found that naive B cells, plasma cells, and resting memory CD4 + T cells were associated with good prognosis in the high IRGPI group. In contrast, memory B cells, M0 macrophages, and M2 macrophages were associated with poor prognosis in the high IRGPI subgroup (**Figure 6B**). Finally, the Limma package of R was used to investigate the differences in immune cell function between the high- and low-risk groups. Our results showed that there were differences in B cells, CCR, CD8+ T cells, and DCs between the two groups (**Figure 6C**). Further, survival analysis of functionally differentiated cells showed that B cells, DCs, T cell co-stimulation, etc., were significantly different between the two groups (**Supplementary Figure 4**).

### Clinical Correlation Analysis and ICI Treatment of Different IRGPI Subgroups

The chi-square test was used to observe differences in clinical traits among different IRGPI subgroups. Our results revealed differences in age, pathological stage, T stage, N stage, M stage, ER, and HER2 statuses, but not in the PR status. In addition, there were differences between BC immunotypes and IRGPI subgroups (**Supplementary Figure 5**). Further analysis found that in TNBC patients, PD-L1 expression was different between high and low risk groups (*P*<0.05), and IRGPI score was positively correlated with PD-L1 expression (**Supplementary Figure 6**).

**Figure 7:**
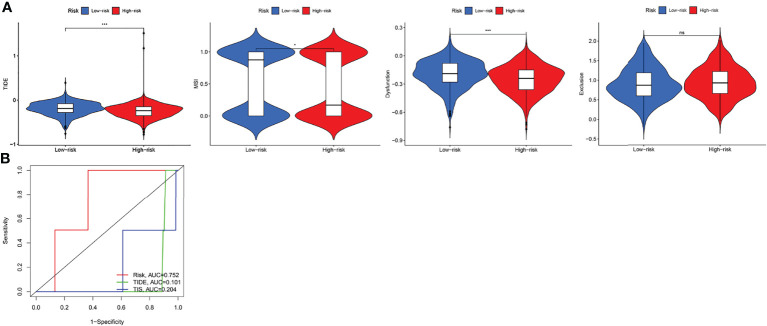
The prognostic value of IRGPI in ICI therapy. **(A)** TIDE, MSI, and T-cell exclusion and dysfunction score in different IRGPI subgroups. The scores between the two IRGPI subgroups were compared through the Wilcoxon test (ns, not significant, *P <0.05, ***P <0.001). **(B)** ROC analysis of IRGPI, TIS, and TIDE on OS at 5-year follow-up in breast cancer cohort.

TIDE evaluates the potential clinical efficacy of immunotherapy in different IRGPI subgroups. The higher the TIDE score, the greater the possibility of immune evasion and the worse the ICI effect. The IRGPI-high subgroup had a low TIDE score, MSI and T cell dysfunction scores (*p* < 0.05). However, there was no difference in T cell exclusion between the two subgroups (**Figure 7A**). In addition, comparison of the IRGPI, TIDE, and TIS models showed that the AUCs of IRGPI were better during the 5-year follow-up (**Figure 7B**).

## Discussion

The role of immune regulation in the pathogenesis, development, and prognosis of BC has been widely recognized (12). Immunotherapy is expected to become an innovative cancer treatment due to its high specificity. BC is highly heterogeneous, with a low overall response rate to ICI treatment, and a lack of immune genetic markers capable of predicting the response and OS to immunotherapy. Prognostic markers constructed based on IRGs have been proven to aid in assessing the OS of a variety of cancers, including head and neck squamous cell carcinoma (HNSCC), renal cell carcinoma (RCC), and bladder cancer (13–15). However, the mechanism underlying the complex interactions between immune genes, BC, and the host immune responses is poorly understood. In this study, we screened BC-related immune genes and conducted WGCNA; selected 196 genes in the modules most relevant to BC; established IRGPI through univariate, LASSO, and multivariate regression analyses; and performed validation studies. Our results showed that IRGPI could be used as an independent prognostic factor for BC.

IRGPI consists of six genes: SOCS3, TCF7L2, and TSLP, which are protective factors, and NPR3, ANO6, and HMGB3 which are risk factors. SOCS3 gene is a tumor suppressor that plays a role in the regulation of various signaling pathways and immune molecules, thereby preventing malignant proliferation, invasion, and metastasis (16, 17). SOCS3 silences the expression of its downstream gene, C-myc, and reduces the possibility of tumor occurrence (18). In BC, high SOCS3 expression is associated with a better prognosis, whereas low SOCS3 is associated with poor prognosis (19). The TCF7L2 protein, a key transcriptional effector of the Wnt/β-catenin signaling pathway, which regulates gene expression, is associated with numerous diseases such as cancer, diabetes, and small intestinal Crohn’s disease (20). TCF7L2 has been shown to be part of a super-enhancer (SE) called EphA2-SE, which promotes the expression of genes that define the properties of healthy and diseased cells (21). In BC, the rs7903146-T allele of the most common TCF7L2 variant was significantly associated with lymph node involvement. TSLP is a widely studied cytokine with various roles in different cancers (22). TSLP plays a significant role in the tumor microenvironment leading to tumor progression and promotion of angiogenesis and metastasis in both solid tumors, such as cervical, ovarian, and pancreatic cancer; and liquid tumors, such as lymphoma and acute lymphoblastic leukemia (23–25). In addition, TSLP-mediated antitumor effects have also been reported in skin, colon cancer, and BC (26, 27). NPR3 is involved in the pathogenesis of cancer and can act as both a tumor suppressor or promoter in some types of cancer. Upregulation of NPR3 promotes the proliferation of colorectal cancer cells and is associated with poor prognosis. In osteosarcoma, NPR3 acts as a tumor suppressor. In BC, NP3R can be used as a prognostic marker (28). Moreover, ANO6 is involved in the pathogenesis of many diseases, such as cancer, hemorrhagic disease, and bone dysplasia (29). In glioma, high expression of ANO6 is associated with a lower survival rate suggesting that differential expression of ANO6 can be used as an independent prognostic factor in BC (30). HMGB3 is mainly expressed in embryonic and bone marrow hematopoietic stem cells but not in normal adult tissues. The upregulation of HMGB3 can promote tumorigenesis and chemotherapy resistance through various mechanisms (31). It is highly expressed in a variety of cancers, such as BC, gastric adenocarcinoma, lung cancer, and bladder cancer, and is associated with advanced tumors and low survival rates (32, 33). These results are consistent with those of our study, thereby confirming the reliability of our results. Therefore, considering the regulatory role of these six immune-related genes, our IRGPI can be used as a potential biomarker of BC prognosis.

GSEA analysis revealed biological differences between different IRGPI subsets of genes. The analysis showed that there were more enriched immune response-related pathways in the low-IRGPI group than in the high-IRGP1 group, and that the differences between these two groups may be caused by the complexity of the tumor immune microenvironment. Furthermore, analysis of immune cell infiltration in subgroups showed that the degree of immune infiltration in the low-IRGPI group was significantly higher than that in the high-IRGPI group. This was reflected in the content of immune cells. The low-IRGPI subgroup was rich in natural immune cell infiltrates, including B cells, T cells, natural killer cells, and mast cells. Studies have shown that these immune cells are associated with good prognosis of tumor patients. The high-IRGPI group was rich in M0 and M2 macrophages. M2 macrophages, a major subtype of macrophages found in most tumors, are associated with chronic inflammation and promote tumorigenesis, development of an aggressive phenotype, and poor prognosis in breast, bladder, ovarian, stomach, and prostate cancers (34, 35). This suggests that the high-IRGPI group is characterized by immunosuppression and active tumor progression, which is consistent with our survival results.

Next, we explore the relationship between IRGPI and known predictive biomarkers for immunotherapy, such as PD-L1. By comparing the AUC values of IRGPI and PD-L1, the predictive power of IRGPI was better than that of PD-L1 expression. In TNBC, PD-L1 expression was higher in the high-risk group and IRGPI score was positively correlated with PD-L1 expression. In addition, through clinical correlation and ICI benefit analyses of different IRGPI subgroups, we found that IRGPI showed specificity in distinguishing age, TNM stage, ER, and HER2 statuses, and that the high-IRGPI subgroup had low TIDE, MSI, and T cell dysfunction scores. TIDE can be used to identify two immune escape mechanisms that induce T cell dysfunction in tumors with high CTL invasion and prevent T cell invasion in tumors with low CTL levels. In melanoma studies, TIDE was found to be more accurate than other biomarkers, such as PD-L1 levels and mutant load, in the prognosis of first-line anti-PD1 or anti-CTLA4 antibody therapy (36). By comparing the predictive function of IRGPI with TIDE, we found that IRGPI may have a better predictive ability in long-term follow-ups.

In conclusion, for effective BC prognosis, we established an IRGPI comprising six immune-related genes. The IRGPI subgroup can distinguish the immune and molecular characteristics and clinical features of BC and provide a plan for monitoring the long-term treatment for BC.

## Data Availability Statement

The datasets presented in this study can be found in online repositories. The names of the repository/repositories and accession number(s) can be found in the article/**Supplementary Material**.

## Author Contributions

CS and YY conceived and designed the study. RL and FX performed data analysis. RL contributed analysis tools. YY and XK were the major contributors in writing the manuscript. All authors contributed to the article and approved the submitted version.

## Funding

This work was supported by the National Natural Science Foundation of China (81973677; 82174222) and Shandong Province Natural Science Foundation (ZR2021LZY015, ZR2021MH343).

## Conflict of Interest

The authors declare that the research was conducted in the absence of any commercial or financial relationships that could be construed as a potential conflict of interest.

## Publisher’s Note

All claims expressed in this article are solely those of the authors and do not necessarily represent those of their affiliated organizations, or those of the publisher, the editors and the reviewers. Any product that may be evaluated in this article, or claim that may be made by its manufacturer, is not guaranteed or endorsed by the publisher.
