# Supplementary material for: Development of a Novel Immune-Related Gene Prognostic Index for Breast Cancer
Source: Front Immunol. 2022 Apr 26;13:845093. doi: 10.3389/fimmu.2022.845093 (PMC9086776; doi:10.3389/fimmu.2022.845093)
Supplement: Supplementary file 1 [file DataSheet_1.zip › Data Sheet 1 (1)/Supplementary/Supplementary Table 2.docx]

**Supplementary Table 2: Hub immune-related genes KEGG terms**

| **ID** | **Description** | **pvalue** |
| --- | --- | --- |
| hsa04060 | Cytokine-cytokine receptor interaction | 2.37E-19 |
| hsa04061 | Viral protein interaction with cytokine and cytokine receptor | 5.41E-13 |
| hsa04630 | JAK-STAT signaling pathway | 1.49E-08 |
| hsa04062 | Chemokine signaling pathway | 3.25E-08 |
| hsa04014 | Ras signaling pathway | 1.17E-07 |
| hsa04668 | TNF signaling pathway | 2.48E-07 |
| hsa04010 | MAPK signaling pathway | 2.49E-07 |
| hsa04151 | PI3K-Akt signaling pathway | 3.51E-07 |
| hsa05144 | Malaria | 4.32E-07 |
| hsa04015 | Rap1 signaling pathway | 6.69E-07 |
| hsa04080 | Neuroactive ligand-receptor interaction | 1.14E-06 |
| hsa05218 | Melanoma | 1.20E-06 |
| hsa05323 | Rheumatoid arthritis | 1.79E-06 |
| hsa04020 | Calcium signaling pathway | 4.25E-06 |
| hsa04360 | Axon guidance | 1.23E-05 |
| hsa04933 | AGE-RAGE signaling pathway in diabetic complications | 2.42E-05 |
| hsa04920 | Adipocytokine signaling pathway | 5.57E-05 |
| hsa05417 | Lipid and atherosclerosis | 7.86E-05 |
| hsa04657 | IL-17 signaling pathway | 8.78E-05 |
| hsa03320 | PPAR signaling pathway | 0.000102 |
| hsa05224 | Breast cancer | 0.000139 |
| hsa01521 | EGFR tyrosine kinase inhibitor resistance | 0.000147 |
| hsa05142 | Chagas disease | 0.000165 |
| hsa05207 | Chemical carcinogenesis - receptor activation | 0.000259 |
| hsa04979 | Cholesterol metabolism | 0.000404 |
| hsa05167 | Kaposi sarcoma-associated herpesvirus infection | 0.000414 |
| hsa04935 | Growth hormone synthesis, secretion and action | 0.000526 |
| hsa04510 | Focal adhesion | 0.00057 |
| hsa05226 | Gastric cancer | 0.00067 |
| hsa05133 | Pertussis | 0.000689 |
| hsa04923 | Regulation of lipolysis in adipocytes | 0.000749 |
| hsa04932 | Non-alcoholic fatty liver disease | 0.000911 |
| hsa04024 | cAMP signaling pathway | 0.001212 |
| hsa05161 | Hepatitis B | 0.001277 |
| hsa04012 | ErbB signaling pathway | 0.001342 |
| hsa04610 | Complement and coagulation cascades | 0.001342 |
| hsa04211 | Longevity regulating pathway | 0.001755 |
| hsa04936 | Alcoholic liver disease | 0.001854 |
| hsa05171 | Coronavirus disease - COVID-19 | 0.001979 |
| hsa05205 | Proteoglycans in cancer | 0.002279 |
| hsa04152 | AMPK signaling pathway | 0.00241 |
| hsa04917 | Prolactin signaling pathway | 0.002415 |
| hsa05120 | Epithelial cell signaling in Helicobacter pylori infection | 0.002415 |
| hsa05223 | Non-small cell lung cancer | 0.002787 |
| hsa04621 | NOD-like receptor signaling pathway | 0.003271 |
| hsa05166 | Human T-cell leukemia virus 1 infection | 0.004207 |
| hsa05202 | Transcriptional misregulation in cancer | 0.004429 |
| hsa05163 | Human cytomegalovirus infection | 0.004654 |
| hsa04928 | Parathyroid hormone synthesis, secretion and action | 0.004709 |
| hsa05134 | Legionellosis | 0.005023 |
| hsa04659 | Th17 cell differentiation | 0.005216 |
| hsa04931 | Insulin resistance | 0.005216 |
| hsa04066 | HIF-1 signaling pathway | 0.005484 |
| hsa05162 | Measles | 0.005911 |
| hsa05418 | Fluid shear stress and atherosclerosis | 0.005911 |
| hsa05221 | Acute myeloid leukemia | 0.009913 |
| hsa04350 | TGF-beta signaling pathway | 0.010221 |
| hsa04810 | Regulation of actin cytoskeleton | 0.010537 |
| hsa04924 | Renin secretion | 0.011182 |
| hsa05211 | Renal cell carcinoma | 0.011182 |
| hsa05215 | Prostate cancer | 0.011828 |
| hsa04930 | Type II diabetes mellitus | 0.012276 |
| hsa01522 | Endocrine resistance | 0.0124 |
| hsa05231 | Choline metabolism in cancer | 0.0124 |
| hsa04380 | Osteoclast differentiation | 0.012795 |
| hsa04926 | Relaxin signaling pathway | 0.013313 |
| hsa04068 | FoxO signaling pathway | 0.014394 |
| hsa05146 | Amoebiasis | 0.014888 |
| hsa04672 | Intestinal immune network for IgA production | 0.015237 |
| hsa04064 | NF-kappa B signaling pathway | 0.016253 |
| hsa05220 | Chronic myeloid leukemia | 0.016489 |
