# Supplementary material for: Development of a Novel Immune-Related Gene Prognostic Index for Breast Cancer
Source: Front Immunol. 2022 Apr 26;13:845093. doi: 10.3389/fimmu.2022.845093 (PMC9086776; doi:10.3389/fimmu.2022.845093)
Supplement: Supplementary file 1 [file DataSheet_1.zip › Data Sheet 1 (1)/Supplementary/Supplementary Table1.docx]

**Supplementary Table 1: Hub immune-related genes GO terms**

| **ONTOLOGY** | **Description** | ***P*-value** |
| --- | --- | --- |
| BP | cell chemotaxis | 3.30E-32 |
| BP | myeloid leukocyte migration | 6.52E-25 |
| BP | epithelial cell proliferation | 2.58E-23 |
| BP | regulation of epithelial cell proliferation | 3.70E-22 |
| BP | ERK1 and ERK2 cascade | 3.74E-22 |
| BP | regulation of ERK1 and ERK2 cascade | 1.09E-19 |
| BP | leukocyte chemotaxis | 1.12E-19 |
| BP | regulation of inflammatory response | 2.37E-19 |
| BP | mononuclear cell migration | 3.37E-19 |
| BP | response to chemokine | 3.94E-19 |
| BP | cellular response to chemokine | 3.94E-19 |
| BP | regulation of endothelial cell proliferation | 1.01E-18 |
| BP | regulation of chemotaxis | 1.24E-18 |
| BP | chemokine-mediated signaling pathway | 1.53E-18 |
| BP | negative regulation of response to external stimulus | 2.00E-18 |
| BP | endothelial cell proliferation | 5.76E-18 |
| BP | monocyte chemotaxis | 1.16E-17 |
| BP | muscle cell proliferation | 1.33E-17 |
| BP | positive regulation of epithelial cell proliferation | 2.29E-17 |
| BP | regulation of leukocyte migration | 2.85E-17 |
| BP | positive regulation of ERK1 and ERK2 cascade | 4.89E-17 |
| BP | negative regulation of locomotion | 5.08E-17 |
| BP | ameboidal-type cell migration | 3.53E-16 |
| BP | smooth muscle cell proliferation | 5.65E-16 |
| BP | calcium ion homeostasis | 8.91E-16 |
| BP | granulocyte chemotaxis | 9.31E-16 |
| BP | positive regulation of cell adhesion | 1.22E-15 |
| BP | granulocyte migration | 1.50E-15 |
| BP | cellular calcium ion homeostasis | 3.39E-15 |
| BP | regulation of smooth muscle cell proliferation | 6.48E-15 |
| BP | epithelial cell migration | 8.79E-15 |
| BP | epithelium migration | 1.07E-14 |
| BP | divalent inorganic cation homeostasis | 1.11E-14 |
| BP | neutrophil chemotaxis | 1.29E-14 |
| BP | tissue migration | 1.49E-14 |
| BP | protein kinase B signaling | 1.55E-14 |
| BP | positive regulation of cytosolic calcium ion concentration | 2.64E-14 |
| BP | positive regulation of endothelial cell proliferation | 2.79E-14 |
| BP | cellular divalent inorganic cation homeostasis | 2.85E-14 |
| BP | negative regulation of cellular component movement | 5.30E-14 |
| BP | regulation of epithelial cell migration | 7.04E-14 |
| BP | response to lipopolysaccharide | 8.01E-14 |
| BP | negative regulation of cell migration | 1.05E-13 |
| BP | response to peptide hormone | 1.25E-13 |
| BP | regulation of cell-cell adhesion | 1.40E-13 |
| BP | regulation of lipid metabolic process | 1.43E-13 |
| BP | negative regulation of immune system process | 1.51E-13 |
| BP | peptidyl-tyrosine phosphorylation | 1.54E-13 |
| BP | peptidyl-tyrosine modification | 1.85E-13 |
| BP | positive chemotaxis | 2.08E-13 |
| BP | endothelial cell migration | 2.16E-13 |
| BP | neutrophil migration | 2.26E-13 |
| BP | regulation of cytosolic calcium ion concentration | 2.59E-13 |
| BP | negative regulation of cell motility | 2.76E-13 |
| BP | response to molecule of bacterial origin | 2.94E-13 |
| BP | cellular extravasation | 3.79E-13 |
| BP | regulation of developmental growth | 4.23E-13 |
| BP | regulation of leukocyte cell-cell adhesion | 8.59E-13 |
| BP | negative regulation of leukocyte migration | 1.32E-12 |
| BP | positive regulation of epithelial cell migration | 4.96E-12 |
| BP | positive regulation of protein kinase B signaling | 4.96E-12 |
| BP | regulation of endothelial cell migration | 5.15E-12 |
| BP | response to interleukin-1 | 6.94E-12 |
| BP | leukocyte cell-cell adhesion | 7.25E-12 |
| BP | negative regulation of defense response | 7.52E-12 |
| BP | positive regulation of phosphatidylinositol 3-kinase signaling | 1.09E-11 |
| BP | negative regulation of inflammatory response | 1.38E-11 |
| BP | regulation of protein kinase B signaling | 1.67E-11 |
| BP | epithelial cell apoptotic process | 2.11E-11 |
| BP | fat cell differentiation | 2.26E-11 |
| BP | cellular response to peptide | 2.76E-11 |
| BP | response to alcohol | 5.44E-11 |
| BP | regulation of cellular extravasation | 5.64E-11 |
| BP | positive regulation of leukocyte cell-cell adhesion | 5.84E-11 |
| BP | regulation of phosphatidylinositol 3-kinase signaling | 7.29E-11 |
| BP | phosphatidylinositol 3-kinase signaling | 8.94E-11 |
| BP | regulation of vasculature development | 9.12E-11 |
| BP | neuron death | 9.63E-11 |
| BP | positive regulation of cell-cell adhesion | 1.14E-10 |
| BP | sprouting angiogenesis | 1.15E-10 |
| BP | positive regulation of endothelial cell migration | 1.23E-10 |
| BP | vascular process in circulatory system | 1.25E-10 |
| BP | regulation of lipid localization | 1.72E-10 |
| BP | phosphatidylinositol-mediated signaling | 2.17E-10 |
| BP | positive regulation of peptidyl-tyrosine phosphorylation | 2.34E-10 |
| BP | positive regulation of chemotaxis | 2.47E-10 |
| BP | inositol lipid-mediated signaling | 2.95E-10 |
| BP | negative regulation of cell adhesion | 3.54E-10 |
| BP | lymphocyte migration | 3.57E-10 |
| BP | regulation of peptidyl-tyrosine phosphorylation | 3.72E-10 |
| BP | regulation of cellular response to growth factor stimulus | 3.74E-10 |
| BP | positive regulation of smooth muscle cell proliferation | 3.93E-10 |
| BP | regulation of angiogenesis | 4.52E-10 |
| BP | regulation of anatomical structure size | 4.85E-10 |
| BP | regulation of blood pressure | 6.41E-10 |
| BP | positive regulation of lipid metabolic process | 6.82E-10 |
| BP | cellular response to interleukin-1 | 8.11E-10 |
| BP | negative regulation of growth | 1.00E-09 |
| BP | cell growth | 1.04E-09 |
| BP | endothelial cell chemotaxis | 1.13E-09 |
| BP | lymphocyte chemotaxis | 1.26E-09 |
| BP | negative regulation of chemotaxis | 1.26E-09 |
| BP | negative regulation of developmental growth | 1.41E-09 |
| BP | transmembrane receptor protein serine/threonine kinase signaling pathway | 1.44E-09 |
| BP | positive regulation of cytokine production | 1.45E-09 |
| BP | lipid storage | 1.48E-09 |
| BP | ossification | 1.74E-09 |
| BP | regulation of leukocyte tethering or rolling | 2.06E-09 |
| BP | positive regulation of lipid localization | 2.16E-09 |
| BP | regulation of cell growth | 2.17E-09 |
| BP | regulation of protein binding | 2.64E-09 |
| BP | muscle tissue development | 2.65E-09 |
| BP | regulation of pri-miRNA transcription by RNA polymerase II | 2.80E-09 |
| BP | response to interferon-gamma | 2.83E-09 |
| BP | cellular response to lipopolysaccharide | 2.83E-09 |
| BP | regulation of phospholipase activity | 3.12E-09 |
| BP | positive regulation of small molecule metabolic process | 3.28E-09 |
| BP | pri-miRNA transcription by RNA polymerase II | 3.36E-09 |
| BP | regulation of neuron death | 3.60E-09 |
| BP | cellular response to biotic stimulus | 3.70E-09 |
| BP | temperature homeostasis | 3.81E-09 |
| BP | axon extension involved in axon guidance | 3.96E-09 |
| BP | neuron projection extension involved in neuron projection guidance | 3.96E-09 |
| BP | regulation of leukocyte adhesion to vascular endothelial cell | 3.96E-09 |
| BP | cellular response to vascular endothelial growth factor stimulus | 4.14E-09 |
| BP | regulation of epithelial cell apoptotic process | 4.40E-09 |
| BP | morphogenesis of a branching epithelium | 4.43E-09 |
| BP | leukocyte adhesion to vascular endothelial cell | 4.77E-09 |
| BP | positive regulation of angiogenesis | 5.14E-09 |
| BP | positive regulation of vasculature development | 5.14E-09 |
| BP | cellular response to interferon-gamma | 5.95E-09 |
| BP | regulation of lipase activity | 6.16E-09 |
| BP | cellular response to molecule of bacterial origin | 6.36E-09 |
| BP | positive regulation of anion transport | 7.53E-09 |
| BP | positive regulation of pri-miRNA transcription by RNA polymerase II | 7.68E-09 |
| BP | platelet-derived growth factor receptor signaling pathway | 7.85E-09 |
| BP | regulation of binding | 8.33E-09 |
| BP | urogenital system development | 8.91E-09 |
| BP | morphogenesis of a branching structure | 1.13E-08 |
| BP | response to steroid hormone | 1.44E-08 |
| BP | positive regulation of inflammatory response | 1.72E-08 |
| BP | response to ketone | 1.80E-08 |
| BP | leukocyte tethering or rolling | 2.17E-08 |
| BP | organ growth | 2.38E-08 |
| BP | regulation of mononuclear cell migration | 2.58E-08 |
| BP | positive regulation of sterol transport | 2.78E-08 |
| BP | positive regulation of cholesterol transport | 2.78E-08 |
| BP | second-messenger-mediated signaling | 2.95E-08 |
| BP | regulation of MAP kinase activity | 2.95E-08 |
| BP | organic hydroxy compound transport | 3.15E-08 |
| BP | regulation of chemokine production | 3.36E-08 |
| BP | regulation of axon extension involved in axon guidance | 3.52E-08 |
| BP | blood vessel endothelial cell migration | 4.46E-08 |
| BP | cardiac muscle tissue growth | 4.64E-08 |
| BP | regulation of leukocyte chemotaxis | 5.34E-08 |
| BP | positive regulation of leukocyte activation | 5.34E-08 |
| BP | positive regulation of T cell activation | 5.90E-08 |
| BP | positive regulation of cholesterol efflux | 6.08E-08 |
| BP | regulation of protein serine/threonine kinase activity | 6.13E-08 |
| BP | chemokine production | 6.32E-08 |
| BP | renal system development | 7.32E-08 |
| BP | regulation of transmembrane receptor protein serine/threonine kinase signaling pathway | 7.35E-08 |
| BP | receptor-mediated endocytosis | 7.72E-08 |
| BP | positive regulation of cell activation | 8.17E-08 |
| BP | regulation of cell-matrix adhesion | 8.89E-08 |
| BP | response to estradiol | 8.89E-08 |
| BP | positive regulation of cell-matrix adhesion | 9.16E-08 |
| BP | regulation of lipid transport | 1.02E-07 |
| BP | positive regulation of secretion | 1.02E-07 |
| BP | heart growth | 1.03E-07 |
| BP | developmental cell growth | 1.05E-07 |
| BP | eosinophil migration | 1.11E-07 |
| BP | muscle cell migration | 1.14E-07 |
| BP | response to reactive oxygen species | 1.17E-07 |
| BP | regulation of lipid storage | 1.23E-07 |
| BP | positive regulation of fatty acid metabolic process | 1.25E-07 |
| BP | regeneration | 1.33E-07 |
| BP | regulation of phosphatidylinositol 3-kinase activity | 1.42E-07 |
| BP | response to oxygen levels | 1.49E-07 |
| BP | negative regulation of macrophage derived foam cell differentiation | 1.49E-07 |
| BP | glucose homeostasis | 1.53E-07 |
| BP | negative regulation of neuron death | 1.59E-07 |
| BP | adenylate cyclase-modulating G protein-coupled receptor signaling pathway | 1.62E-07 |
| BP | carbohydrate homeostasis | 1.62E-07 |
| BP | positive regulation of leukocyte migration | 1.68E-07 |
| BP | regulation of actin filament-based process | 1.73E-07 |
| BP | calcium-mediated signaling | 1.90E-07 |
| BP | striated muscle tissue development | 2.03E-07 |
| BP | regulation of small molecule metabolic process | 2.04E-07 |
| BP | regulation of actin cytoskeleton organization | 2.12E-07 |
| BP | regulation of cell development | 2.24E-07 |
| BP | skeletal system development | 2.31E-07 |
| BP | regulation of lipid biosynthetic process | 2.53E-07 |
| BP | positive regulation of developmental growth | 2.60E-07 |
| BP | positive regulation of lipid transport | 2.79E-07 |
| BP | response to decreased oxygen levels | 2.91E-07 |
| BP | kidney development | 3.03E-07 |
| BP | connective tissue development | 3.17E-07 |
| BP | bone mineralization | 3.30E-07 |
| BP | endothelial cell apoptotic process | 3.56E-07 |
| BP | regulation of cholesterol efflux | 3.60E-07 |
| BP | smooth muscle cell migration | 3.76E-07 |
| BP | cold-induced thermogenesis | 3.77E-07 |
| BP | regulation of cold-induced thermogenesis | 3.77E-07 |
| BP | cellular response to peptide hormone stimulus | 3.78E-07 |
| BP | mesenchyme development | 4.16E-07 |
| BP | activation of protein kinase activity | 4.28E-07 |
| BP | lipid transport | 4.61E-07 |
| BP | positive regulation of growth | 5.16E-07 |
| BP | positive regulation of neurogenesis | 5.44E-07 |
| BP | rhythmic process | 5.65E-07 |
| BP | regulation of nervous system development | 5.71E-07 |
| BP | regulation of axonogenesis | 6.07E-07 |
| BP | regulation of lipid kinase activity | 6.43E-07 |
| BP | regulation of cellular ketone metabolic process | 6.47E-07 |
| BP | negative regulation of cell growth | 6.47E-07 |
| BP | vascular endothelial growth factor signaling pathway | 6.68E-07 |
| BP | positive regulation of ossification | 6.68E-07 |
| BP | adaptive thermogenesis | 6.92E-07 |
| BP | regulation of sterol transport | 7.19E-07 |
| BP | regulation of cholesterol transport | 7.19E-07 |
| BP | regulation of cardiac muscle tissue growth | 7.19E-07 |
| BP | transcription initiation from RNA polymerase II promoter | 7.25E-07 |
| BP | osteoblast differentiation | 7.41E-07 |
| BP | regulation of carbohydrate biosynthetic process | 7.94E-07 |
| BP | positive regulation of cell projection organization | 8.05E-07 |
| BP | positive regulation of nervous system development | 8.22E-07 |
| BP | positive regulation of endocytosis | 8.67E-07 |
| BP | positive regulation of cold-induced thermogenesis | 8.67E-07 |
| BP | hemostasis | 8.69E-07 |
| BP | negative regulation of epithelial cell apoptotic process | 8.91E-07 |
| BP | cellular response to chemical stress | 9.02E-07 |
| BP | regulation of macrophage derived foam cell differentiation | 9.22E-07 |
| BP | response to hypoxia | 9.37E-07 |
| BP | regulation of neurogenesis | 9.37E-07 |
| BP | regulation of fatty acid metabolic process | 9.46E-07 |
| BP | extracellular matrix organization | 9.84E-07 |
| BP | animal organ regeneration | 9.97E-07 |
| BP | extracellular structure organization | 1.02E-06 |
| BP | cell-matrix adhesion | 1.05E-06 |
| BP | neuron apoptotic process | 1.05E-06 |
| BP | regulation of osteoblast differentiation | 1.05E-06 |
| BP | biomineral tissue development | 1.08E-06 |
| BP | regulation of tumor necrosis factor production | 1.08E-06 |
| BP | external encapsulating structure organization | 1.09E-06 |
| BP | mesenchymal cell differentiation | 1.10E-06 |
| BP | positive regulation of protein serine/threonine kinase activity | 1.14E-06 |
| BP | positive regulation of secretion by cell | 1.17E-06 |
| BP | response to oxidative stress | 1.19E-06 |
| BP | interleukin-6 production | 1.22E-06 |
| BP | tumor necrosis factor production | 1.22E-06 |
| BP | biomineralization | 1.22E-06 |
| BP | positive regulation of blood vessel endothelial cell migration | 1.23E-06 |
| BP | positive regulation of lymphocyte activation | 1.26E-06 |
| BP | eosinophil chemotaxis | 1.28E-06 |
| BP | intracellular receptor signaling pathway | 1.34E-06 |
| BP | regulation of tumor necrosis factor superfamily cytokine production | 1.38E-06 |
| BP | cell-substrate adhesion | 1.41E-06 |
| BP | phospholipase C-activating G protein-coupled receptor signaling pathway | 1.44E-06 |
| BP | reproductive structure development | 1.49E-06 |
| BP | regulation of heart growth | 1.51E-06 |
| BP | axon guidance | 1.52E-06 |
| BP | receptor signaling pathway via JAK-STAT | 1.55E-06 |
| BP | negative regulation of protein phosphorylation | 1.57E-06 |
| BP | maintenance of location | 1.57E-06 |
| BP | neuron projection guidance | 1.59E-06 |
| BP | negative regulation of phosphorylation | 1.59E-06 |
| BP | response to tumor necrosis factor | 1.63E-06 |
| BP | reproductive system development | 1.64E-06 |
| BP | tumor necrosis factor superfamily cytokine production | 1.65E-06 |
| BP | response to hydrogen peroxide | 1.71E-06 |
| BP | regulation of fat cell differentiation | 1.83E-06 |
| BP | negative regulation of cytokine production | 1.88E-06 |
| BP | gland development | 1.94E-06 |
| BP | gliogenesis | 1.96E-06 |
| BP | acute inflammatory response | 1.99E-06 |
| BP | nephron development | 2.09E-06 |
| BP | regulation of T cell activation | 2.13E-06 |
| BP | regulation of smooth muscle cell migration | 2.22E-06 |
| BP | cardiac muscle tissue development | 2.23E-06 |
| BP | cholesterol transport | 2.32E-06 |
| BP | penile erection | 2.40E-06 |
| BP | regulation of endothelial cell apoptotic process | 2.50E-06 |
| BP | DNA-templated transcription, initiation | 2.54E-06 |
| BP | receptor signaling pathway via STAT | 2.60E-06 |
| BP | positive regulation of cell development | 2.60E-06 |
| BP | macrophage derived foam cell differentiation | 2.67E-06 |
| BP | foam cell differentiation | 2.67E-06 |
| BP | gland morphogenesis | 2.70E-06 |
| BP | negative regulation of lipid storage | 2.83E-06 |
| BP | regulation of ossification | 2.91E-06 |
| BP | T cell activation | 2.99E-06 |
| BP | positive regulation of bone mineralization | 3.12E-06 |
| BP | regulation of neuron projection development | 3.13E-06 |
| BP | positive regulation of chemokine production | 3.15E-06 |
| BP | regulation of multicellular organism growth | 3.15E-06 |
| BP | cellular ketone metabolic process | 3.16E-06 |
| BP | cellular response to tumor necrosis factor | 3.30E-06 |
| BP | axon extension | 3.37E-06 |
| BP | body fluid secretion | 3.50E-06 |
| BP | negative regulation of mononuclear cell migration | 3.58E-06 |
| BP | regulation of cell-substrate adhesion | 3.62E-06 |
| BP | hormone metabolic process | 3.62E-06 |
| BP | cardiac chamber morphogenesis | 3.62E-06 |
| BP | semaphorin-plexin signaling pathway | 3.64E-06 |
| BP | cellular response to oxidative stress | 3.71E-06 |
| BP | ovulation cycle | 3.94E-06 |
| BP | gluconeogenesis | 4.16E-06 |
| BP | cellular response to amyloid-beta | 4.23E-06 |
| BP | negative regulation of smooth muscle cell proliferation | 4.39E-06 |
| BP | regulation of blood vessel endothelial cell migration | 4.43E-06 |
| BP | cartilage development | 4.45E-06 |
| BP | growth hormone receptor signaling pathway | 4.49E-06 |
| BP | regulation of endothelial cell chemotaxis | 4.49E-06 |
| BP | regulation of vascular permeability | 4.89E-06 |
| BP | cholesterol efflux | 4.89E-06 |
| BP | developmental growth involved in morphogenesis | 5.01E-06 |
| BP | regulation of DNA-binding transcription factor activity | 5.10E-06 |
| BP | hexose biosynthetic process | 5.36E-06 |
| BP | regulation of response to cytokine stimulus | 5.46E-06 |
| BP | positive regulation of cell-substrate adhesion | 5.51E-06 |
| BP | cellular response to growth hormone stimulus | 5.56E-06 |
| BP | glomerulus vasculature development | 5.56E-06 |
| BP | activation of MAPK activity | 5.60E-06 |
| BP | BMP signaling pathway | 5.60E-06 |
| BP | regulation of axon extension | 5.82E-06 |
| BP | peptide secretion | 5.82E-06 |
| BP | sterol transport | 5.90E-06 |
| BP | positive regulation of MAP kinase activity | 6.27E-06 |
| BP | cell migration involved in sprouting angiogenesis | 6.31E-06 |
| BP | positive regulation of osteoblast differentiation | 6.67E-06 |
| BP | positive regulation of phagocytosis | 6.67E-06 |
| BP | negative regulation of axon extension involved in axon guidance | 6.83E-06 |
| BP | renal system vasculature development | 6.83E-06 |
| BP | kidney vasculature development | 6.83E-06 |
| BP | regulation of phagocytosis | 6.83E-06 |
| BP | regulation of actin filament organization | 6.94E-06 |
| BP | regulation of interleukin-6 production | 7.04E-06 |
| BP | positive regulation of defense response | 7.09E-06 |
| BP | cellular response to reactive oxygen species | 7.44E-06 |
| BP | interleukin-1 production | 7.68E-06 |
| BP | response to monosaccharide | 7.73E-06 |
| BP | stress-activated protein kinase signaling cascade | 7.80E-06 |
| BP | negative regulation of secretion | 7.87E-06 |
| BP | positive regulation of phagocytosis, engulfment | 7.98E-06 |
| BP | negative regulation of leukocyte adhesion to vascular endothelial cell | 7.98E-06 |
| BP | positive regulation of membrane invagination | 7.98E-06 |
| BP | response to activity | 8.12E-06 |
| BP | regulation of tube diameter | 8.19E-06 |
| BP | blood vessel diameter maintenance | 8.19E-06 |
| BP | regulation of monocyte chemotaxis | 8.31E-06 |
| BP | regulation of systemic arterial blood pressure mediated by a chemical signal | 8.43E-06 |
| BP | brown fat cell differentiation | 8.43E-06 |
| BP | negative chemotaxis | 8.43E-06 |
| BP | response to insulin | 8.43E-06 |
| BP | monosaccharide biosynthetic process | 8.64E-06 |
| BP | protein secretion | 8.64E-06 |
| BP | platelet degranulation | 8.73E-06 |
| BP | regulation of tube size | 8.73E-06 |
| BP | regulation of protein secretion | 8.77E-06 |
| BP | establishment of protein localization to extracellular region | 8.92E-06 |
| BP | heart morphogenesis | 9.26E-06 |
| BP | regulation of immune effector process | 9.35E-06 |
| BP | regulation of cellular component size | 9.52E-06 |
| BP | regulation of gluconeogenesis | 9.58E-06 |
| BP | positive regulation of biomineral tissue development | 9.58E-06 |
| BP | regulation of response to wounding | 9.78E-06 |
| BP | digestive system process | 1.00E-05 |
| BP | myeloid cell differentiation | 1.01E-05 |
| BP | response to mechanical stimulus | 1.03E-05 |
| BP | regulation of wound healing | 1.05E-05 |
| BP | positive regulation of biomineralization | 1.09E-05 |
| BP | steroid metabolic process | 1.11E-05 |
| BP | protein localization to extracellular region | 1.12E-05 |
| BP | response to BMP | 1.15E-05 |
| BP | cellular response to BMP stimulus | 1.15E-05 |
| BP | positive regulation of cartilage development | 1.20E-05 |
| BP | signal release | 1.23E-05 |
| BP | regulation of endocytosis | 1.24E-05 |
| BP | regulation of organ growth | 1.25E-05 |
| BP | response to extracellular stimulus | 1.30E-05 |
| BP | humoral immune response | 1.30E-05 |
| BP | positive regulation of lymphocyte proliferation | 1.34E-05 |
| BP | response to glucocorticoid | 1.34E-05 |
| BP | phagocytosis | 1.35E-05 |
| BP | response to retinoic acid | 1.35E-05 |
| BP | regulation of cell division | 1.41E-05 |
| BP | carbohydrate biosynthetic process | 1.42E-05 |
| BP | positive regulation of mononuclear cell proliferation | 1.43E-05 |
| BP | response to prostaglandin | 1.43E-05 |
| BP | regulation of extent of cell growth | 1.45E-05 |
| BP | positive regulation of fatty acid oxidation | 1.50E-05 |
| BP | positive regulation of nitric-oxide synthase biosynthetic process | 1.50E-05 |
| BP | regulation of phagocytosis, engulfment | 1.50E-05 |
| BP | positive regulation of endothelial cell chemotaxis | 1.50E-05 |
| BP | regulation of bone mineralization | 1.55E-05 |
| BP | SMAD protein signal transduction | 1.55E-05 |
| BP | apoptotic cell clearance | 1.55E-05 |
| BP | regulation of multi-organism process | 1.55E-05 |
| BP | response to amyloid-beta | 1.55E-05 |
| BP | digestion | 1.60E-05 |
| BP | negative regulation of cell development | 1.64E-05 |
| BP | regulation of glucose transmembrane transport | 1.69E-05 |
| BP | blood coagulation | 1.71E-05 |
| BP | tissue remodeling | 1.72E-05 |
| BP | negative regulation of transport | 1.74E-05 |
| BP | vasculogenesis | 1.84E-05 |
| BP | positive regulation of axonogenesis | 1.84E-05 |
| BP | endocrine process | 1.84E-05 |
| BP | regulation of cytokine-mediated signaling pathway | 1.90E-05 |
| BP | macrophage migration | 1.94E-05 |
| BP | regulation of membrane invagination | 1.98E-05 |
| BP | regulation of myeloid cell differentiation | 1.99E-05 |
| BP | regulation of fibroblast proliferation | 2.00E-05 |
| BP | coagulation | 2.00E-05 |
| BP | hormone secretion | 2.03E-05 |
| BP | anatomical structure maturation | 2.10E-05 |
| BP | branching morphogenesis of an epithelial tube | 2.13E-05 |
| BP | blood vessel endothelial cell proliferation involved in sprouting angiogenesis | 2.17E-05 |
| BP | positive regulation of cellular carbohydrate metabolic process | 2.17E-05 |
| BP | fibroblast proliferation | 2.17E-05 |
| BP | aging | 2.18E-05 |
| BP | negative regulation of cellular response to growth factor stimulus | 2.18E-05 |
| BP | negative regulation of cell projection organization | 2.20E-05 |
| BP | regulation of cellular carbohydrate metabolic process | 2.25E-05 |
| BP | regulation of stress-activated MAPK cascade | 2.31E-05 |
| BP | regulation of hormone metabolic process | 2.32E-05 |
| BP | receptor internalization | 2.33E-05 |
| BP | female sex differentiation | 2.33E-05 |
| BP | regulation of anion transmembrane transport | 2.33E-05 |
| BP | response to carbohydrate | 2.38E-05 |
| BP | neural crest cell migration | 2.41E-05 |
| BP | regulation of peptide secretion | 2.50E-05 |
| BP | regulation of ketone biosynthetic process | 2.57E-05 |
| BP | regulation of stress-activated protein kinase signaling cascade | 2.66E-05 |
| BP | positive regulation of cell migration involved in sprouting angiogenesis | 2.70E-05 |
| BP | negative regulation of endothelial cell apoptotic process | 2.70E-05 |
| BP | positive regulation of protein binding | 2.76E-05 |
| BP | positive regulation of leukocyte proliferation | 2.80E-05 |
| BP | negative regulation of epithelial cell migration | 2.82E-05 |
| BP | hormone transport | 2.85E-05 |
| BP | developmental maturation | 2.89E-05 |
| BP | insulin-like growth factor receptor signaling pathway | 3.12E-05 |
| BP | axonogenesis | 3.16E-05 |
| BP | regulation of tyrosine phosphorylation of STAT protein | 3.22E-05 |
| BP | positive regulation of lipid biosynthetic process | 3.22E-05 |
| BP | leukocyte migration involved in inflammatory response | 3.28E-05 |
| BP | regulation of cholesterol storage | 3.28E-05 |
| BP | cardiac chamber development | 3.28E-05 |
| BP | negative regulation of cell-cell adhesion | 3.34E-05 |
| BP | response to corticosteroid | 3.46E-05 |
| BP | regulation of amine transport | 3.48E-05 |
| BP | regulation of systemic arterial blood pressure by hormone | 3.59E-05 |
| BP | regulation of hemopoiesis | 3.63E-05 |
| BP | regulation of interleukin-1 production | 3.85E-05 |
| BP | positive regulation of phospholipase activity | 3.99E-05 |
| BP | production of molecular mediator involved in inflammatory response | 4.04E-05 |
| BP | tyrosine phosphorylation of STAT protein | 4.04E-05 |
| BP | regulation of supramolecular fiber organization | 4.05E-05 |
| BP | negative regulation of leukocyte chemotaxis | 4.12E-05 |
| BP | copulation | 4.12E-05 |
| BP | positive regulation of multi-organism process | 4.12E-05 |
| BP | regulation of response to biotic stimulus | 4.16E-05 |
| BP | alcohol metabolic process | 4.42E-05 |
| BP | regulation of systemic arterial blood pressure | 4.67E-05 |
| BP | mating | 4.70E-05 |
| BP | regulation of neuron apoptotic process | 4.75E-05 |
| BP | glomerulus development | 4.81E-05 |
| BP | regulation of reproductive process | 4.92E-05 |
| BP | mesonephros development | 5.01E-05 |
| BP | I-kappaB phosphorylation | 5.10E-05 |
| BP | cholesterol storage | 5.10E-05 |
| BP | regulation of hormone biosynthetic process | 5.10E-05 |
| BP | nitric-oxide synthase biosynthetic process | 5.10E-05 |
| BP | regulation of nitric-oxide synthase biosynthetic process | 5.10E-05 |
| BP | response to growth hormone | 5.35E-05 |
| BP | negative regulation of protein kinase activity | 5.66E-05 |
| BP | cardiac septum morphogenesis | 5.76E-05 |
| BP | in utero embryonic development | 5.93E-05 |
| BP | lung development | 5.97E-05 |
| BP | C21-steroid hormone metabolic process | 6.06E-05 |
| BP | response to interleukin-7 | 6.06E-05 |
| BP | cellular response to interleukin-7 | 6.06E-05 |
| BP | regulation of neuroinflammatory response | 6.06E-05 |
| BP | amine transport | 6.17E-05 |
| BP | regulation of biomineral tissue development | 6.17E-05 |
| BP | regulation of GTPase activity | 6.23E-05 |
| BP | sympathetic nervous system development | 6.25E-05 |
| BP | cell proliferation involved in kidney development | 6.25E-05 |
| BP | neuron projection extension | 6.26E-05 |
| BP | pathway-restricted SMAD protein phosphorylation | 6.30E-05 |
| BP | regulation of blood coagulation | 6.86E-05 |
| BP | negative regulation of axonogenesis | 6.86E-05 |
| BP | regulation of cartilage development | 6.86E-05 |
| BP | T cell migration | 6.86E-05 |
| BP | vascular endothelial growth factor receptor signaling pathway | 7.06E-05 |
| BP | regulation of biomineralization | 7.06E-05 |
| BP | respiratory tube development | 7.20E-05 |
| BP | positive regulation of transmembrane transport | 7.20E-05 |
| BP | positive regulation of ion transmembrane transport | 7.20E-05 |
| BP | regulation of hemostasis | 7.47E-05 |
| BP | receptor metabolic process | 7.54E-05 |
| BP | negative regulation of endothelial cell migration | 7.54E-05 |
| BP | negative regulation of transferase activity | 7.56E-05 |
| BP | cellular response to transforming growth factor beta stimulus | 7.57E-05 |
| BP | positive regulation of leukocyte adhesion to vascular endothelial cell | 7.58E-05 |
| BP | genitalia development | 7.70E-05 |
| BP | negative regulation of neuron projection development | 8.04E-05 |
| BP | regulation of cytokine production involved in inflammatory response | 8.13E-05 |
| BP | positive regulation of binding | 8.26E-05 |
| BP | positive regulation of T cell proliferation | 8.59E-05 |
| BP | negative regulation of axon extension | 8.64E-05 |
| BP | regulation of p38MAPK cascade | 8.64E-05 |
| BP | cytokine production involved in inflammatory response | 8.82E-05 |
| BP | positive regulation of tyrosine phosphorylation of STAT protein | 8.82E-05 |
| BP | regulation of cell-substrate junction organization | 8.82E-05 |
| BP | negative regulation of transmembrane receptor protein serine/threonine kinase signaling pathway | 8.93E-05 |
| BP | response to dietary excess | 9.10E-05 |
| BP | response to prostaglandin E | 9.10E-05 |
| BP | olefinic compound biosynthetic process | 9.10E-05 |
| BP | positive regulation of alcohol biosynthetic process | 9.10E-05 |
| BP | smooth muscle contraction | 9.15E-05 |
| BP | response to nutrient levels | 9.26E-05 |
| BP | response to transforming growth factor beta | 9.34E-05 |
| BP | negative regulation of neuron apoptotic process | 9.41E-05 |
| BP | regulation of cell size | 9.44E-05 |
| BP | positive regulation of phospholipase C activity | 9.66E-05 |
| BP | neuroinflammatory response | 9.66E-05 |
| BP | regulation of hormone secretion | 0.00010357 |
| BP | regulation of coagulation | 0.00010359 |
| BP | endothelium development | 0.00010431 |
| BP | negative regulation of hydrolase activity | 0.00010465 |
| BP | negative regulation of anion transport | 0.00010649 |
| BP | stress-activated MAPK cascade | 0.00010714 |
| BP | positive regulation of glucose transmembrane transport | 0.00010779 |
| BP | positive regulation of smooth muscle cell migration | 0.00010779 |
| BP | positive regulation of glycoprotein biosynthetic process | 0.00010826 |
| BP | chondrocyte differentiation | 0.00011032 |
| BP | actin cytoskeleton reorganization | 0.00011032 |
| BP | interleukin-8 production | 0.00011032 |
| BP | sex differentiation | 0.00011082 |
| BP | striated muscle cell proliferation | 0.00011202 |
| BP | forebrain development | 0.00011242 |
| BP | negative regulation of secretion by cell | 0.00011541 |
| BP | acute-phase response | 0.00011991 |
| BP | lactation | 0.00011991 |
| BP | positive regulation of fibroblast proliferation | 0.00011991 |
| BP | autonomic nervous system development | 0.00011991 |
| BP | regulation of phospholipase C activity | 0.00011991 |
| BP | positive regulation of lipase activity | 0.00012099 |
| BP | positive regulation of GTPase activity | 0.00012407 |
| BP | mononuclear cell differentiation | 0.00012407 |
| BP | leukocyte proliferation | 0.00012423 |
| BP | regulation of pattern recognition receptor signaling pathway | 0.00012451 |
| BP | integrin activation | 0.00012782 |
| BP | positive regulation of positive chemotaxis | 0.00012782 |
| BP | negative regulation of kinase activity | 0.00013089 |
| BP | negative regulation of cholesterol storage | 0.00013144 |
| BP | endothelial cell chemotaxis to fibroblast growth factor | 0.00013144 |
| BP | negative regulation of multicellular organism growth | 0.00013144 |
| BP | regulation of endothelial cell chemotaxis to fibroblast growth factor | 0.00013144 |
| BP | regulation of extracellular matrix organization | 0.00013304 |
| BP | cellular response to fibroblast growth factor stimulus | 0.00013387 |
| BP | regulation of DNA biosynthetic process | 0.00014014 |
| BP | adenylate cyclase-activating G protein-coupled receptor signaling pathway | 0.00014054 |
| BP | muscle organ development | 0.00014412 |
| BP | regulation of acute inflammatory response | 0.00014725 |
| BP | glial cell proliferation | 0.00014725 |
| BP | interleukin-1 beta production | 0.00014853 |
| BP | protein autophosphorylation | 0.00014861 |
| BP | C21-steroid hormone biosynthetic process | 0.00014982 |
| BP | regulation of positive chemotaxis | 0.00014982 |
| BP | negative regulation of cytokine-mediated signaling pathway | 0.00015137 |
| BP | negative regulation of ion transport | 0.0001528 |
| BP | response to glucose | 0.00015714 |
| BP | glucose transmembrane transport | 0.00015733 |
| BP | response to temperature stimulus | 0.00015966 |
| BP | p38MAPK cascade | 0.00016259 |
| BP | regulation of cardiac muscle cell proliferation | 0.00016259 |
| BP | regulation of toll-like receptor signaling pathway | 0.00016274 |
| BP | regulation of receptor signaling pathway via STAT | 0.00016654 |
| BP | positive regulation of glycoprotein metabolic process | 0.00017443 |
| BP | positive regulation of carbohydrate metabolic process | 0.00017477 |
| BP | negative regulation of ERK1 and ERK2 cascade | 0.00017477 |
| BP | respiratory system development | 0.00017733 |
| BP | response to fibroblast growth factor | 0.00017814 |
| BP | response to vitamin E | 0.00017932 |
| BP | cell chemotaxis to fibroblast growth factor | 0.00017932 |
| BP | regulation of cell chemotaxis to fibroblast growth factor | 0.00017932 |
| BP | hexose transmembrane transport | 0.00018627 |
| BP | negative regulation of vasculature development | 0.00018657 |
| BP | vasoconstriction | 0.0001875 |
| BP | response to hexose | 0.00019197 |
| BP | neural crest cell development | 0.00020094 |
| BP | positive regulation of reproductive process | 0.00020094 |
| BP | monosaccharide transmembrane transport | 0.00020785 |
| BP | I-kappaB kinase/NF-kappaB signaling | 0.00021063 |
| BP | extracellular matrix disassembly | 0.00021513 |
| BP | negative regulation of response to cytokine stimulus | 0.00021513 |
| BP | hormone-mediated signaling pathway | 0.00021579 |
| BP | bone development | 0.00021579 |
| BP | positive regulation of cellular catabolic process | 0.00023002 |
| BP | positive regulation of stress-activated MAPK cascade | 0.00023141 |
| BP | carbohydrate transmembrane transport | 0.00023141 |
| BP | dendritic cell migration | 0.0002322 |
| BP | heart valve morphogenesis | 0.00023648 |
| BP | negative regulation of fat cell differentiation | 0.00023648 |
| BP | complement activation, lectin pathway | 0.00023724 |
| BP | negative regulation of platelet-derived growth factor receptor signaling pathway | 0.00023724 |
| BP | positive regulation of hormone metabolic process | 0.00023724 |
| BP | bone mineralization involved in bone maturation | 0.00023724 |
| BP | commissural neuron axon guidance | 0.00023724 |
| BP | stem cell development | 0.00024588 |
| BP | cardiac cell development | 0.00024588 |
| BP | regulation of vascular associated smooth muscle cell proliferation | 0.00024588 |
| BP | vascular associated smooth muscle cell proliferation | 0.00024588 |
| BP | regulation of leukocyte proliferation | 0.00024979 |
| BP | positive regulation of stress-activated protein kinase signaling cascade | 0.00025707 |
| BP | mesenchymal cell development | 0.0002625 |
| BP | odontogenesis of dentin-containing tooth | 0.0002625 |
| BP | interleukin-7-mediated signaling pathway | 0.00026572 |
| BP | negative regulation of cardiac muscle tissue growth | 0.00026572 |
| BP | negative regulation of heart growth | 0.00026572 |
| BP | heart trabecula morphogenesis | 0.00026572 |
| BP | positive regulation of cell-substrate junction organization | 0.00026572 |
| BP | regulation of glucose metabolic process | 0.00027074 |
| BP | endothelial cell differentiation | 0.00027074 |
| BP | regulation of I-kappaB kinase/NF-kappaB signaling | 0.00027575 |
| BP | defense response to Gram-negative bacterium | 0.00027999 |
| BP | ureteric bud development | 0.00029838 |
| BP | positive regulation of interleukin-6 production | 0.00029838 |
| BP | positive regulation of cell division | 0.00029838 |
| BP | positive regulation of vasoconstriction | 0.00030258 |
| BP | peptide hormone secretion | 0.00030394 |
| BP | activation of transmembrane receptor protein tyrosine kinase activity | 0.00030602 |
| BP | positive regulation of catecholamine secretion | 0.00030602 |
| BP | positive regulation of urine volume | 0.00030602 |
| BP | positive regulation of renal sodium excretion | 0.00030602 |
| BP | white fat cell differentiation | 0.00030602 |
| BP | heart trabecula formation | 0.00030602 |
| BP | semaphorin-plexin signaling pathway involved in neuron projection guidance | 0.00030602 |
| BP | regulation of glycoprotein biosynthetic process | 0.00030683 |
| BP | positive regulation of catabolic process | 0.0003146 |
| BP | regulation of steroid metabolic process | 0.00031526 |
| BP | positive regulation of tumor necrosis factor production | 0.0003177 |
| BP | mesonephric epithelium development | 0.0003177 |
| BP | mesonephric tubule development | 0.0003177 |
| BP | multi-multicellular organism process | 0.0003252 |
| BP | negative regulation of protein transport | 0.00033135 |
| BP | positive regulation of interleukin-8 production | 0.00033349 |
| BP | muscle system process | 0.00033635 |
| BP | regulation of apoptotic signaling pathway | 0.00033895 |
| BP | regulation of renal system process | 0.00034297 |
| BP | regulation of carbohydrate metabolic process | 0.00034933 |
| BP | regulation of peptidase activity | 0.00035155 |
| BP | bone remodeling | 0.00035929 |
| BP | lipopolysaccharide-mediated signaling pathway | 0.00036185 |
| BP | cardiac muscle cell proliferation | 0.00036185 |
| BP | neural crest cell differentiation | 0.00038162 |
| BP | cellular response to hydrogen peroxide | 0.00038162 |
| BP | positive regulation of tumor necrosis factor superfamily cytokine production | 0.00038162 |
| BP | response to ethanol | 0.00038359 |
| BP | neutrophil homeostasis | 0.00038646 |
| BP | growth hormone receptor signaling pathway via JAK-STAT | 0.00038646 |
| BP | physiological muscle hypertrophy | 0.00038708 |
| BP | physiological cardiac muscle hypertrophy | 0.00038708 |
| BP | positive regulation of phosphatidylinositol 3-kinase activity | 0.00038708 |
| BP | regulation of fatty acid oxidation | 0.00038708 |
| BP | positive regulation of amine transport | 0.00038708 |
| BP | response to lipoprotein particle | 0.00038708 |
| BP | cell growth involved in cardiac muscle cell development | 0.00038708 |
| BP | cellular response to external stimulus | 0.00038872 |
| BP | regulation of smooth muscle contraction | 0.00039201 |
| BP | regulation of macrophage activation | 0.00039201 |
| BP | glucose metabolic process | 0.00040202 |
| BP | negative regulation of establishment of protein localization | 0.00040239 |
| BP | positive regulation of leukocyte chemotaxis | 0.00040502 |
| BP | regulation of BMP signaling pathway | 0.00040502 |
| BP | regulation of steroid biosynthetic process | 0.00040502 |
| BP | regulation of T cell proliferation | 0.00042038 |
| BP | excretion | 0.00042402 |
| BP | negative regulation of hormone secretion | 0.00042402 |
| BP | negative regulation of myeloid cell differentiation | 0.00042952 |
| BP | positive regulation of neuron death | 0.00042952 |
| BP | circadian rhythm | 0.00043073 |
| BP | cellular response to lipoprotein particle stimulus | 0.00043509 |
| BP | lymphocyte differentiation | 0.00043698 |
| BP | response to drug | 0.00044799 |
| BP | feeding behavior | 0.00045517 |
| BP | heart valve development | 0.00045797 |
| BP | regulation of vasoconstriction | 0.00045797 |
| BP | animal organ formation | 0.00045797 |
| BP | response to fatty acid | 0.00045797 |
| BP | positive regulation of anion transmembrane transport | 0.00045797 |
| BP | multicellular organism growth | 0.00046328 |
| BP | positive regulation of keratinocyte proliferation | 0.00047932 |
| BP | progesterone metabolic process | 0.00047932 |
| BP | lipoprotein transport | 0.00047932 |
| BP | positive regulation of gluconeogenesis | 0.00047932 |
| BP | response to epinephrine | 0.00047932 |
| BP | metanephric glomerulus development | 0.00047932 |
| BP | kidney epithelium development | 0.00048513 |
| BP | energy homeostasis | 0.00048721 |
| BP | regulation of glycoprotein metabolic process | 0.00049392 |
| BP | mammary gland development | 0.0005078 |
| BP | extrinsic apoptotic signaling pathway | 0.00050995 |
| BP | negative regulation of neurogenesis | 0.0005313 |
| BP | positive regulation of mononuclear cell migration | 0.00053196 |
| BP | regulation of lymphocyte migration | 0.00053196 |
| BP | regulation of innate immune response | 0.00053808 |
| BP | cardiac septum development | 0.00053933 |
| BP | cardiac muscle hypertrophy | 0.00053933 |
| BP | negative regulation of smooth muscle cell migration | 0.00054362 |
| BP | positive regulation of DNA-binding transcription factor activity | 0.00054413 |
| BP | positive regulation of immune effector process | 0.00054481 |
| BP | regulation of interleukin-8 production | 0.00056992 |
| BP | sensory system development | 0.00057149 |
| BP | ventricular septum development | 0.00057215 |
| BP | regulation of focal adhesion assembly | 0.00057215 |
| BP | regulation of cell-substrate junction assembly | 0.00057215 |
| BP | regulation of lymphocyte proliferation | 0.00058161 |
| BP | female genitalia development | 0.00058537 |
| BP | heat generation | 0.00058537 |
| BP | lipoprotein localization | 0.00058537 |
| BP | positive regulation of myeloid leukocyte cytokine production involved in immune response | 0.00058537 |
| BP | negative regulation of organ growth | 0.00060452 |
| BP | ventricular septum morphogenesis | 0.00060452 |
| BP | positive regulation of lipid kinase activity | 0.00060452 |
| BP | cellular hormone metabolic process | 0.00060704 |
| BP | regulation of mononuclear cell proliferation | 0.00062042 |
| BP | positive regulation of production of molecular mediator of immune response | 0.00063512 |
| BP | striated muscle hypertrophy | 0.00063512 |
| BP | sensory perception of pain | 0.00063512 |
| BP | regulation of reactive oxygen species biosynthetic process | 0.00063512 |
| BP | cell killing | 0.00063966 |
| BP | negative regulation of interleukin-6 production | 0.00065933 |
| BP | negative regulation of nervous system development | 0.00066213 |
| BP | negative regulation of leukocyte cell-cell adhesion | 0.00066213 |
| BP | positive regulation of interleukin-10 production | 0.00067011 |
| BP | macrophage chemotaxis | 0.00067011 |
| BP | negative regulation of striated muscle cell differentiation | 0.00067011 |
| BP | regulation of actin cytoskeleton reorganization | 0.00067011 |
| BP | cellular response to insulin stimulus | 0.0006826 |
| BP | negative regulation of MAPK cascade | 0.00068799 |
| BP | lymphangiogenesis | 0.0007053 |
| BP | positive regulation of chondrocyte differentiation | 0.0007053 |
| BP | negative regulation of vascular permeability | 0.0007053 |
| BP | negative regulation of lipase activity | 0.0007053 |
| BP | negative regulation of neuroinflammatory response | 0.0007053 |
| BP | muscle hypertrophy | 0.00070597 |
| BP | regulation of receptor signaling pathway via JAK-STAT | 0.00070597 |
| BP | negative regulation of small molecule metabolic process | 0.00070597 |
| BP | regulation of plasma lipoprotein particle levels | 0.00070597 |
| BP | negative regulation of lipid localization | 0.00070648 |
| BP | positive regulation of I-kappaB kinase/NF-kappaB signaling | 0.00071324 |
| BP | positive regulation of axon extension | 0.00074058 |
| BP | negative regulation of DNA biosynthetic process | 0.00074058 |
| BP | macrophage activation | 0.00074361 |
| BP | actin filament organization | 0.00074734 |
| BP | catecholamine transport | 0.0007561 |
| BP | regulation of interleukin-1 beta production | 0.00078278 |
| BP | regulation of leukocyte differentiation | 0.00078588 |
| BP | regulation of actin polymerization or depolymerization | 0.00079349 |
| BP | telencephalon development | 0.00079747 |
| BP | nerve development | 0.00080827 |
| BP | positive regulation of cardiac muscle tissue growth | 0.00081614 |
| BP | positive regulation of epithelial cell apoptotic process | 0.00081614 |
| BP | regulation of peptidyl-serine phosphorylation | 0.00081731 |
| BP | regulation of actin filament length | 0.00082179 |
| BP | reactive oxygen species metabolic process | 0.00082992 |
| BP | retina vasculature development in camera-type eye | 0.0008398 |
| BP | response to purine-containing compound | 0.00085154 |
| BP | cardiocyte differentiation | 0.00085154 |
| BP | fatty acid oxidation | 0.00086589 |
| BP | negative regulation of hemopoiesis | 0.00086589 |
| BP | regulation of signaling receptor activity | 0.00088084 |
| BP | negative regulation of protein serine/threonine kinase activity | 0.00088689 |
| BP | positive regulation of glucose metabolic process | 0.00089697 |
| BP | cell-substrate junction organization | 0.00090992 |
| BP | negative regulation of tumor necrosis factor production | 0.00092063 |
| BP | regulation of production of molecular mediator of immune response | 0.00092338 |
| BP | negative regulation of angiogenesis | 0.00092338 |
| BP | regulation of protein-containing complex assembly | 0.00093921 |
| BP | positive regulation of peptidyl-serine phosphorylation | 0.00095564 |
| BP | positive regulation of transmembrane receptor protein serine/threonine kinase signaling pathway | 0.00095564 |
| BP | fatty acid metabolic process | 0.00096748 |
| BP | long-chain fatty acid transport | 0.00098097 |
| BP | astrocyte differentiation | 0.00098097 |
| BP | positive regulation of response to wounding | 0.00098097 |
| BP | astrocyte development | 0.00098329 |
| BP | ketone biosynthetic process | 0.00098329 |
| BP | positive regulation of steroid biosynthetic process | 0.00098955 |
| BP | regulation of renal sodium excretion | 0.00098955 |
| BP | cellular response to leptin stimulus | 0.00098955 |
| BP | positive regulation of actin cytoskeleton reorganization | 0.00098955 |
| BP | negative regulation of blood vessel morphogenesis | 0.00099992 |
| BP | regulation of reactive oxygen species metabolic process | 0.00100916 |
| BP | cholesterol metabolic process | 0.00104001 |
| BP | carbohydrate transport | 0.00104001 |
| BP | pancreas development | 0.00104421 |
| BP | negative regulation of tumor necrosis factor superfamily cytokine production | 0.00104421 |
| BP | regulation of blood circulation | 0.00105398 |
| BP | ovulation cycle process | 0.00107528 |
| BP | trabecula morphogenesis | 0.00107528 |
| BP | positive regulation of signaling receptor activity | 0.00107528 |
| BP | regulation of T cell migration | 0.00107528 |
| BP | DNA biosynthetic process | 0.0010787 |
| BP | fatty acid transport | 0.00108136 |
| BP | negative regulation of immune response | 0.00108136 |
| BP | lipid oxidation | 0.0011035 |
| BP | T cell proliferation | 0.00111488 |
| BP | regulation of cell junction assembly | 0.00111488 |
| BP | regulation of cell shape | 0.00112399 |
| BP | regulation of mitochondrion organization | 0.00112399 |
| BP | regulation of muscle system process | 0.00113839 |
| BP | ossification involved in bone maturation | 0.00115517 |
| BP | type B pancreatic cell proliferation | 0.00115517 |
| BP | branching involved in salivary gland morphogenesis | 0.00115517 |
| BP | cellular response to prostaglandin stimulus | 0.00115517 |
| BP | cell junction disassembly | 0.00115517 |
| BP | negative regulation of organelle organization | 0.00115843 |
| BP | negative regulation of cellular carbohydrate metabolic process | 0.00117314 |
| BP | positive regulation of heart growth | 0.00117314 |
| BP | regulation of alcohol biosynthetic process | 0.00117968 |
| BP | epithelial to mesenchymal transition | 0.0012132 |
| BP | positive regulation of neuron projection development | 0.0012132 |
| BP | T cell differentiation | 0.00123983 |
| BP | negative regulation of peptide secretion | 0.0012521 |
| BP | fibroblast growth factor receptor signaling pathway | 0.0012683 |
| BP | response to progesterone | 0.00127708 |
| BP | cardiac muscle cell differentiation | 0.00132721 |
| BP | negative regulation of MAP kinase activity | 0.00132775 |
| BP | cardiac muscle cell development | 0.00132775 |
| BP | regulation of cell migration involved in sprouting angiogenesis | 0.00132775 |
| BP | response to metal ion | 0.00132908 |
| BP | detection of temperature stimulus | 0.00133729 |
| BP | regulation of urine volume | 0.00133729 |
| BP | renal sodium excretion | 0.00133729 |
| BP | lymph vessel morphogenesis | 0.00133729 |
| BP | peptidyl-tyrosine autophosphorylation | 0.00133729 |
| BP | alcohol biosynthetic process | 0.00135732 |
| BP | hexose metabolic process | 0.00138631 |
| BP | calcineurin-mediated signaling | 0.00138728 |
| BP | regulation of complement activation | 0.00138819 |
| BP | mucopolysaccharide metabolic process | 0.00138819 |
| BP | transforming growth factor beta receptor signaling pathway | 0.00139615 |
| BP | platelet activation | 0.00140822 |
| BP | stem cell differentiation | 0.00142503 |
| BP | secondary alcohol metabolic process | 0.0014606 |
| BP | myeloid leukocyte differentiation | 0.00148608 |
| BP | monoamine transport | 0.0014891 |
| BP | microglial cell activation | 0.00150396 |
| BP | response to cold | 0.00150396 |
| BP | regulation of blood vessel endothelial cell proliferation involved in sprouting angiogenesis | 0.00150396 |
| BP | positive regulation of vascular associated smooth muscle cell proliferation | 0.00150396 |
| BP | negative regulation of binding | 0.00151449 |
| BP | calcium-mediated signaling using intracellular calcium source | 0.00153647 |
| BP | regulation of excretion | 0.00153647 |
| BP | response to hyperoxia | 0.00153647 |
| BP | trabecula formation | 0.00153647 |
| BP | cellular response to low-density lipoprotein particle stimulus | 0.00153647 |
| BP | negative regulation of epithelial cell proliferation | 0.00156992 |
| BP | renal system process | 0.00158402 |
| BP | positive regulation of cell growth | 0.00162692 |
| BP | positive regulation of pathway-restricted SMAD protein phosphorylation | 0.00162729 |
| BP | keratinocyte proliferation | 0.00162729 |
| BP | positive regulation of receptor signaling pathway via STAT | 0.00162729 |
| BP | insulin secretion | 0.00162955 |
| BP | negative regulation of peptidase activity | 0.00167603 |
| BP | lipid homeostasis | 0.00168551 |
| BP | epithelial tube morphogenesis | 0.00169107 |
| BP | visual system development | 0.00169554 |
| BP | atrioventricular valve morphogenesis | 0.0017533 |
| BP | regulation of platelet-derived growth factor receptor signaling pathway | 0.0017533 |
| BP | regulation of appetite | 0.0017533 |
| BP | innervation | 0.0017533 |
| BP | regulation of cell growth involved in cardiac muscle cell development | 0.0017533 |
| BP | bone maturation | 0.0017533 |
| BP | regulation of granulocyte chemotaxis | 0.00175749 |
| BP | steroid hormone biosynthetic process | 0.00175749 |
| BP | positive regulation of protein transport | 0.00181547 |
| BP | sterol metabolic process | 0.0018712 |
| BP | response to ischemia | 0.00189474 |
| BP | glial cell migration | 0.00189474 |
| BP | negative regulation of carbohydrate metabolic process | 0.00189474 |
| BP | positive regulation of receptor-mediated endocytosis | 0.00189474 |
| BP | regulation of endopeptidase activity | 0.00195223 |
| BP | regulation of cytokine production involved in immune response | 0.00195524 |
| BP | positive regulation of mitochondrion organization | 0.00195524 |
| BP | positive regulation of JNK cascade | 0.00195524 |
| BP | odontogenesis | 0.00195627 |
| BP | reactive oxygen species biosynthetic process | 0.00195627 |
| BP | dendritic cell chemotaxis | 0.0019883 |
| BP | norepinephrine transport | 0.0019883 |
| BP | response to leptin | 0.0019883 |
| BP | positive regulation of cytokine production involved in inflammatory response | 0.0019883 |
| BP | positive regulation of hormone secretion | 0.00203804 |
| BP | positive regulation of epithelial to mesenchymal transition | 0.00203924 |
| BP | regulation of bone remodeling | 0.00203924 |
| BP | focal adhesion assembly | 0.00205995 |
| BP | actin polymerization or depolymerization | 0.00206625 |
| BP | negative regulation of immune effector process | 0.00212237 |
| BP | digestive tract development | 0.00212237 |
| BP | regulation of tissue remodeling | 0.00216869 |
| BP | branching involved in ureteric bud morphogenesis | 0.00219117 |
| BP | regulation of catecholamine secretion | 0.00219117 |
| BP | outflow tract septum morphogenesis | 0.00224201 |
| BP | atrioventricular valve development | 0.00224201 |
| BP | regulation of lipopolysaccharide-mediated signaling pathway | 0.00224201 |
| BP | negative regulation of chemokine production | 0.00224201 |
| BP | positive regulation of focal adhesion assembly | 0.00224201 |
| BP | regulation of receptor binding | 0.00224201 |
| BP | transport across blood-brain barrier | 0.00228155 |
| BP | striated muscle cell differentiation | 0.00228422 |
| BP | lipid modification | 0.00234192 |
| BP | positive regulation of cytokine production involved in immune response | 0.00235074 |
| BP | response to fungus | 0.00235074 |
| BP | forebrain cell migration | 0.00235074 |
| BP | catecholamine secretion | 0.00235074 |
| BP | vascular transport | 0.00239863 |
| BP | monosaccharide metabolic process | 0.00246081 |
| BP | regulation of JNK cascade | 0.00248629 |
| BP | positive regulation of protein secretion | 0.00248629 |
| BP | lymph vessel development | 0.0025149 |
| BP | negative regulation of amine transport | 0.0025149 |
| BP | myeloid leukocyte cytokine production | 0.0025149 |
| BP | bone growth | 0.0025149 |
| BP | regulation of fatty acid biosynthetic process | 0.00251813 |
| BP | positive regulation of organ growth | 0.00251813 |
| BP | cyclic-nucleotide-mediated signaling | 0.00252002 |
| BP | cellular response to alcohol | 0.00252002 |
| BP | regulation of protein localization to nucleus | 0.00258418 |
| BP | positive regulation of establishment of protein localization | 0.00261007 |
| BP | aorta development | 0.00269352 |
| BP | female pregnancy | 0.00277749 |
| BP | negative regulation of DNA-binding transcription factor activity | 0.00277749 |
| BP | positive regulation of acute inflammatory response | 0.00280746 |
| BP | hair follicle morphogenesis | 0.00280746 |
| BP | inositol phosphate biosynthetic process | 0.00280746 |
| BP | regulation of osteoblast proliferation | 0.00280746 |
| BP | animal organ maturation | 0.00280746 |
| BP | regulation of mitochondrial fission | 0.00280746 |
| BP | energy derivation by oxidation of organic compounds | 0.00284648 |
| BP | complement activation | 0.00286595 |
| BP | regulation of insulin secretion | 0.00286595 |
| BP | regulation of protein import into nucleus | 0.00287711 |
| BP | peptidyl-serine modification | 0.00290875 |
| BP | negative regulation of NF-kappaB transcription factor activity | 0.00291095 |
| BP | cellular response to ketone | 0.00291095 |
| BP | steroid hormone mediated signaling pathway | 0.00300492 |
| BP | digestive system development | 0.00300492 |
| BP | cytokine production involved in immune response | 0.00305048 |
| BP | female gonad development | 0.00305048 |
| BP | regulation of striated muscle cell differentiation | 0.00305048 |
| BP | response to cAMP | 0.00305048 |
| BP | T cell costimulation | 0.00306908 |
| BP | cardiac muscle tissue morphogenesis | 0.00306908 |
| BP | negative regulation of reproductive process | 0.00306908 |
| BP | response to iron ion | 0.00312014 |
| BP | positive regulation of vascular endothelial growth factor production | 0.00312014 |
| BP | positive regulation of lipid storage | 0.00312014 |
| BP | reproductive behavior | 0.00312014 |
| BP | myeloid cell apoptotic process | 0.00312014 |
| BP | negative regulation of receptor signaling pathway via JAK-STAT | 0.00312014 |
| BP | lymphocyte proliferation | 0.00312913 |
| BP | cholesterol homeostasis | 0.00319478 |
| BP | ureteric bud morphogenesis | 0.00326962 |
| BP | sterol homeostasis | 0.00334393 |
| BP | artery development | 0.00334393 |
| BP | regulation of nervous system process | 0.0033526 |
| BP | mononuclear cell proliferation | 0.0033553 |
| BP | salivary gland morphogenesis | 0.00345336 |
| BP | response to increased oxygen levels | 0.00345336 |
| BP | positive regulation of steroid metabolic process | 0.00345336 |
| BP | negative regulation of ATP metabolic process | 0.00345336 |
| BP | liver development | 0.00347489 |
| BP | regulation of humoral immune response | 0.00347489 |
| BP | lymphocyte costimulation | 0.0034789 |
| BP | regulation of nitric oxide biosynthetic process | 0.0034789 |
| BP | regulation of glucose import | 0.0034789 |
| BP | mesonephric tubule morphogenesis | 0.0034789 |
| BP | positive regulation of wound healing | 0.0034789 |
| BP | regulation of protein import | 0.0034789 |
| BP | negative regulation of protein binding | 0.00349803 |
| BP | positive regulation of cell junction assembly | 0.00349803 |
| BP | learning or memory | 0.0035653 |
| BP | cellular response to extracellular stimulus | 0.0035653 |
| BP | cellular carbohydrate metabolic process | 0.00359415 |
| BP | regulation of muscle adaptation | 0.00365717 |
| BP | development of primary female sexual characteristics | 0.00365717 |
| BP | regulation of interleukin-10 production | 0.00369711 |
| BP | regulation of organic acid transport | 0.00369711 |
| BP | heterotypic cell-cell adhesion | 0.00369711 |
| BP | hepaticobiliary system development | 0.0037294 |
| BP | regulation of type 2 immune response | 0.00380754 |
| BP | motor neuron axon guidance | 0.00380754 |
| BP | cGMP-mediated signaling | 0.00380754 |
| BP | positive regulation of filopodium assembly | 0.00380754 |
| BP | positive regulation of cardiac muscle cell proliferation | 0.00380754 |
| BP | positive regulation of animal organ morphogenesis | 0.00380754 |
| BP | positive regulation of T cell migration | 0.00380754 |
| BP | defense response to Gram-positive bacterium | 0.00382144 |
| BP | regulation of nitric oxide metabolic process | 0.00392442 |
| BP | regulation of morphogenesis of an epithelium | 0.00392442 |
| BP | JNK cascade | 0.00398811 |
| BP | antimicrobial humoral response | 0.0039975 |
| BP | regulation of neuron differentiation | 0.00410458 |
| BP | glycosaminoglycan catabolic process | 0.004161 |
| BP | interleukin-10 production | 0.004161 |
| BP | bone resorption | 0.004161 |
| BP | regulation of pathway-restricted SMAD protein phosphorylation | 0.004161 |
| BP | negative regulation of response to biotic stimulus | 0.00416571 |
| BP | cell-substrate junction assembly | 0.00416571 |
| BP | intrinsic apoptotic signaling pathway in response to DNA damage | 0.00416571 |
| BP | regulation of epithelial to mesenchymal transition | 0.00416571 |
| BP | salivary gland development | 0.00418307 |
| BP | epidermis morphogenesis | 0.00418307 |
| BP | regulation of amino acid transport | 0.00418307 |
| BP | cardiac epithelial to mesenchymal transition | 0.00418307 |
| BP | response to endoplasmic reticulum stress | 0.0042968 |
| BP | negative regulation of leukocyte differentiation | 0.00434591 |
| BP | positive regulation of reactive oxygen species metabolic process | 0.00434591 |
| BP | regulation of DNA metabolic process | 0.00438764 |
| BP | positive regulation of gliogenesis | 0.00440704 |
| BP | polyol biosynthetic process | 0.00440704 |
| BP | regulation of actin filament bundle assembly | 0.0045316 |
| BP | negative regulation of endopeptidase activity | 0.00457706 |
| BP | activation of phospholipase C activity | 0.00458033 |
| BP | osteoblast proliferation | 0.00458033 |
| BP | regulation of calcineurin-NFAT signaling cascade | 0.00458033 |
| BP | regulation of neutrophil chemotaxis | 0.00458033 |
| BP | positive regulation of interleukin-1 production | 0.0046627 |
| BP | negative regulation of muscle cell differentiation | 0.0046627 |
| BP | luteinization | 0.0047138 |
| BP | fever generation | 0.0047138 |
| BP | facial nerve development | 0.0047138 |
| BP | facial nerve morphogenesis | 0.0047138 |
| BP | positive regulation of vesicle fusion | 0.0047138 |
| BP | aldosterone metabolic process | 0.0047138 |
| BP | aldosterone biosynthetic process | 0.0047138 |
| BP | positive regulation of interleukin-5 production | 0.0047138 |
| BP | regulation of natural killer cell proliferation | 0.0047138 |
| BP | sterol import | 0.0047138 |
| BP | dorsal aorta development | 0.0047138 |
| BP | maternal behavior | 0.0047138 |
| BP | vagina development | 0.0047138 |
| BP | regulation of gastric acid secretion | 0.0047138 |
| BP | glucagon secretion | 0.0047138 |
| BP | regulation of glucagon secretion | 0.0047138 |
| BP | response to lipoteichoic acid | 0.0047138 |
| BP | cholesterol import | 0.0047138 |
| BP | neutrophil mediated cytotoxicity | 0.0047138 |
| BP | response to interleukin-9 | 0.0047138 |
| BP | cellular response to lipoteichoic acid | 0.0047138 |
| BP | smooth muscle cell chemotaxis | 0.0047138 |
| BP | regulation of glomerular mesangial cell proliferation | 0.0047138 |
| BP | cell proliferation involved in metanephros development | 0.0047138 |
| BP | regulation of steroid hormone biosynthetic process | 0.0047138 |
| BP | type B pancreatic cell apoptotic process | 0.0047138 |
| BP | complement-dependent cytotoxicity | 0.0047138 |
| BP | positive regulation of gonad development | 0.0047138 |
| BP | regulation of male gonad development | 0.0047138 |
| BP | positive regulation of chemokine (C-X-C motif) ligand 2 production | 0.0047138 |
| BP | regulation of nucleocytoplasmic transport | 0.00472286 |
| BP | regulation of intracellular protein transport | 0.00491991 |
| BP | regulation of monooxygenase activity | 0.00492815 |
| BP | negative regulation of innate immune response | 0.00492815 |
| BP | cranial skeletal system development | 0.00492815 |
| BP | eye development | 0.00493112 |
| BP | endocardial cushion morphogenesis | 0.00499965 |
| BP | maintenance of blood-brain barrier | 0.00499965 |
| BP | neuron cellular homeostasis | 0.00499965 |
| BP | regulation of calcineurin-mediated signaling | 0.00499965 |
| BP | negative regulation of receptor signaling pathway via STAT | 0.00499965 |
| BP | positive regulation of fat cell differentiation | 0.00520355 |
| BP | regulation of cell morphogenesis | 0.00521094 |
| BP | positive regulation of cellular protein catabolic process | 0.00521438 |
| BP | muscle cell differentiation | 0.00522201 |
| BP | cell fate commitment | 0.00528214 |
| BP | organic hydroxy compound biosynthetic process | 0.00528214 |
| BP | regulation of receptor-mediated endocytosis | 0.00533104 |
| BP | nephron epithelium development | 0.00533104 |
| BP | positive regulation of peptide secretion | 0.00538364 |
| BP | negative regulation of cell activation | 0.00541603 |
| BP | branching involved in blood vessel morphogenesis | 0.00544138 |
| BP | hindlimb morphogenesis | 0.00544138 |
| BP | wound healing, spreading of cells | 0.00544138 |
| BP | positive regulation of blood pressure | 0.00544138 |
| BP | epiboly involved in wound healing | 0.00544138 |
| BP | negative regulation of cell-substrate adhesion | 0.00548908 |
| BP | killing of cells of other organism | 0.00548908 |
| BP | circadian regulation of gene expression | 0.00548908 |
| BP | muscle tissue morphogenesis | 0.00548908 |
| BP | nephron tubule morphogenesis | 0.00548908 |
| BP | alpha-beta T cell differentiation | 0.00554552 |
| BP | cellular response to steroid hormone stimulus | 0.00556264 |
| BP | positive regulation of protein-containing complex assembly | 0.00566442 |
| BP | natural killer cell proliferation | 0.00572146 |
| BP | suckling behavior | 0.00572146 |
| BP | positive regulation of protein kinase A signaling | 0.00572146 |
| BP | trigeminal nerve development | 0.00572146 |
| BP | positive regulation of interleukin-13 production | 0.00572146 |
| BP | leptin-mediated signaling pathway | 0.00572146 |
| BP | positive regulation of osteoblast proliferation | 0.00572146 |
| BP | positive regulation of MHC class II biosynthetic process | 0.00572146 |
| BP | serotonin uptake | 0.00572146 |
| BP | parental behavior | 0.00572146 |
| BP | glomerular mesangial cell proliferation | 0.00572146 |
| BP | protein localization to cell cortex | 0.00572146 |
| BP | dendritic cell apoptotic process | 0.00572146 |
| BP | synapse pruning | 0.00572146 |
| BP | cellular response to oxidised low-density lipoprotein particle stimulus | 0.00572146 |
| BP | semaphorin-plexin signaling pathway involved in axon guidance | 0.00572146 |
| BP | positive regulation of membrane depolarization | 0.00572146 |
| BP | regulation of matrix metallopeptidase secretion | 0.00572146 |
| BP | matrix metallopeptidase secretion | 0.00572146 |
| BP | regulation of dendritic cell apoptotic process | 0.00572146 |
| BP | positive regulation of proteolysis | 0.0057366 |
| BP | triglyceride metabolic process | 0.00576602 |
| BP | peptidyl-serine phosphorylation | 0.00578024 |
| BP | camera-type eye development | 0.00578024 |
| BP | aminoglycan catabolic process | 0.00578489 |
| BP | regulation of cardiac muscle hypertrophy | 0.00578489 |
| BP | epiboly | 0.00590584 |
| BP | negative regulation of reactive oxygen species biosynthetic process | 0.00590584 |
| BP | positive regulation of response to endoplasmic reticulum stress | 0.00590584 |
| BP | actin filament bundle assembly | 0.00591557 |
| BP | positive regulation of leukocyte differentiation | 0.00591557 |
| BP | positive regulation of hemopoiesis | 0.00591557 |
| BP | regulation of extrinsic apoptotic signaling pathway | 0.00591557 |
| BP | positive regulation of proteasomal protein catabolic process | 0.00599264 |
| BP | negative regulation of catabolic process | 0.00602097 |
| BP | hormone biosynthetic process | 0.00609115 |
| BP | negative regulation of protein secretion | 0.00609115 |
| BP | nephron epithelium morphogenesis | 0.00609115 |
| BP | regulation of cysteine-type endopeptidase activity involved in apoptotic process | 0.006179 |
| BP | regulation of epithelial cell differentiation | 0.00629087 |
| BP | aortic valve development | 0.00639331 |
| BP | type 2 immune response | 0.00639331 |
| BP | vasodilation | 0.00639331 |
| BP | regulation of sensory perception of pain | 0.00639331 |
| BP | glycerolipid metabolic process | 0.00641824 |
| BP | toll-like receptor signaling pathway | 0.00648488 |
| BP | skeletal muscle organ development | 0.00648488 |
| BP | tissue homeostasis | 0.00649197 |
| BP | pattern recognition receptor signaling pathway | 0.00667372 |
| BP | glycosaminoglycan metabolic process | 0.00668321 |
| BP | negative regulation of cytoskeleton organization | 0.00668321 |
| BP | actin filament bundle organization | 0.00668321 |
| BP | regulation of muscle hypertrophy | 0.0067356 |
| BP | response to estrogen | 0.0067356 |
| BP | renal tubule morphogenesis | 0.0067356 |
| BP | nephron morphogenesis | 0.0067356 |
| BP | regulation of angiotensin levels in blood | 0.00681831 |
| BP | angiotensin maturation | 0.00681831 |
| BP | positive regulation of systemic arterial blood pressure | 0.00681831 |
| BP | insemination | 0.00681831 |
| BP | regulation of skeletal muscle satellite cell proliferation | 0.00681831 |
| BP | negative regulation of protein autophosphorylation | 0.00681831 |
| BP | regulation of phospholipase A2 activity | 0.00681831 |
| BP | genitalia morphogenesis | 0.00681831 |
| BP | interleukin-2-mediated signaling pathway | 0.00681831 |
| BP | negative regulation of protein import into nucleus | 0.00681831 |
| BP | estrous cycle | 0.00681831 |
| BP | positive regulation of keratinocyte migration | 0.00681831 |
| BP | negative regulation of amino acid transport | 0.00681831 |
| BP | negative regulation of cell growth involved in cardiac muscle cell development | 0.00681831 |
| BP | regulation of type B pancreatic cell proliferation | 0.00681831 |
| BP | macrophage proliferation | 0.00681831 |
| BP | positive regulation of neuron projection regeneration | 0.00681831 |
| BP | cellular response to epinephrine stimulus | 0.00681831 |
| BP | negative regulation of protein import | 0.00681831 |
| BP | positive regulation of ATP biosynthetic process | 0.00681831 |
| BP | regulation of peptide hormone secretion | 0.00684499 |
| BP | regulation of keratinocyte proliferation | 0.00690409 |
| BP | skeletal muscle tissue regeneration | 0.00690409 |
| BP | regulation of sensory perception | 0.00690409 |
| BP | regulation of response to reactive oxygen species | 0.00690409 |
| BP | positive regulation of lymphocyte migration | 0.00690409 |
| BP | glial cell differentiation | 0.00701952 |
| BP | cardiac ventricle development | 0.00722044 |
| BP | outflow tract morphogenesis | 0.00742367 |
| BP | nitric oxide biosynthetic process | 0.00742367 |
| BP | tissue regeneration | 0.00742367 |
| BP | muscle organ morphogenesis | 0.00742367 |
| BP | mesenchymal cell proliferation | 0.00743843 |
| BP | positive regulation of glucose import | 0.00743843 |
| BP | amyloid-beta clearance | 0.00743843 |
| BP | organic acid transport | 0.00763192 |
| BP | glucose import | 0.00778443 |
| BP | negative regulation of wound healing | 0.00778443 |
| BP | regionalization | 0.00792967 |
| BP | hyaluronan metabolic process | 0.00799658 |
| BP | regulation of digestive system process | 0.00799658 |
| BP | defense response to fungus | 0.00799658 |
| BP | intestinal absorption | 0.00799658 |
| BP | metanephric nephron development | 0.00799658 |
| BP | regulation of macrophage migration | 0.00799658 |
| BP | regulation of neuron migration | 0.00799658 |
| BP | negative regulation of humoral immune response | 0.00800236 |
| BP | mineralocorticoid biosynthetic process | 0.00800236 |
| BP | parturition | 0.00800236 |
| BP | mineralocorticoid metabolic process | 0.00800236 |
| BP | regulation of skeletal muscle cell proliferation | 0.00800236 |
| BP | neuron remodeling | 0.00800236 |
| BP | regulation of prostaglandin biosynthetic process | 0.00800236 |
| BP | regulation of heat generation | 0.00800236 |
| BP | negative regulation of response to food | 0.00800236 |
| BP | negative regulation of appetite | 0.00800236 |
| BP | negative regulation of response to extracellular stimulus | 0.00800236 |
| BP | negative regulation of response to nutrient levels | 0.00800236 |
| BP | response to follicle-stimulating hormone | 0.00800236 |
| BP | primary alcohol biosynthetic process | 0.00800236 |
| BP | interleukin-15-mediated signaling pathway | 0.00800236 |
| BP | positive regulation of inositol phosphate biosynthetic process | 0.00800236 |
| BP | cellular response to interleukin-15 | 0.00800236 |
| BP | cellular response to interleukin-2 | 0.00800236 |
| BP | positive regulation of endoplasmic reticulum unfolded protein response | 0.00800236 |
| BP | regulation of tau-protein kinase activity | 0.00800236 |
| BP | positive regulation of glial cell migration | 0.00800236 |
| BP | regulation of gonad development | 0.00800236 |
| BP | negative regulation of endothelial cell proliferation | 0.00815653 |
| BP | response to nutrient | 0.00819678 |
| BP | regulation of myeloid leukocyte differentiation | 0.00832144 |
| BP | apoptotic mitochondrial changes | 0.00832144 |
| BP | positive regulation of protein ubiquitination | 0.00832144 |
| BP | muscle adaptation | 0.00832144 |
| BP | positive regulation of calcium ion transport | 0.00832144 |
| BP | cell maturation | 0.00843154 |
| BP | negative regulation of blood pressure | 0.00857878 |
| BP | mammary gland morphogenesis | 0.00857878 |
| BP | olefinic compound metabolic process | 0.00861389 |
| BP | muscle cell development | 0.00891549 |
| BP | nitric oxide metabolic process | 0.0089353 |
| BP | positive regulation of cytoskeleton organization | 0.00895138 |
| BP | protein localization to nucleus | 0.00896136 |
| BP | cognition | 0.00914788 |
| BP | regulation of actin filament polymerization | 0.00916479 |
| BP | negative regulation of cell-matrix adhesion | 0.00918524 |
| BP | prostate gland development | 0.00918524 |
| BP | calcineurin-NFAT signaling cascade | 0.00918524 |
| BP | exocrine system development | 0.00918524 |
| BP | T-helper 1 type immune response | 0.00918524 |
| BP | negative regulation of peptide hormone secretion | 0.00918524 |
| BP | negative regulation of intracellular protein transport | 0.00918524 |
| BP | positive regulation of ATP metabolic process | 0.00918524 |
| BP | semi-lunar valve development | 0.00918524 |
| BP | regulation of sequestering of triglyceride | 0.00927168 |
| BP | regulation of macrophage cytokine production | 0.00927168 |
| BP | skeletal muscle satellite cell proliferation | 0.00927168 |
| BP | preganglionic parasympathetic fiber development | 0.00927168 |
| BP | Cdc42 protein signal transduction | 0.00927168 |
| BP | negative regulation of interferon-beta production | 0.00927168 |
| BP | negative regulation of collagen biosynthetic process | 0.00927168 |
| BP | platelet-derived growth factor receptor-beta signaling pathway | 0.00927168 |
| BP | cellular response to increased oxygen levels | 0.00927168 |
| BP | negative regulation of glycolytic process | 0.00927168 |
| BP | negative regulation of smooth muscle contraction | 0.00927168 |
| BP | induction of positive chemotaxis | 0.00927168 |
| BP | regulation of keratinocyte migration | 0.00927168 |
| BP | response to interleukin-2 | 0.00927168 |
| BP | response to interleukin-15 | 0.00927168 |
| BP | G protein-coupled receptor signaling pathway involved in heart process | 0.00927168 |
| BP | mast cell migration | 0.00927168 |
| BP | regulation of cell proliferation involved in kidney development | 0.00927168 |
| BP | regulation of axon guidance | 0.00927168 |
| BP | negative regulation of blood vessel endothelial cell migration | 0.00934225 |
| BP | antimicrobial humoral immune response mediated by antimicrobial peptide | 0.00934225 |
| BP | reactive nitrogen species metabolic process | 0.00934225 |
| BP | endocrine system development | 0.00953377 |
| BP | leukocyte homeostasis | 0.00976109 |
| BP | mitochondrial fission | 0.00981616 |
| BP | endocardial cushion development | 0.00981616 |
| BP | regulation of collagen biosynthetic process | 0.00981616 |
| BP | positive regulation of pattern recognition receptor signaling pathway | 0.00981616 |
| BP | regulation of neutrophil migration | 0.00981616 |
| BP | negative regulation of apoptotic signaling pathway | 0.0098242 |
| BP | Ras protein signal transduction | 0.00990636 |
| BP | aminoglycan metabolic process | 0.00994256 |
| BP | regulation of protein polymerization | 0.0100518 |
| BP | positive regulation of protein catabolic process | 0.0100518 |
| BP | regulation of cation transmembrane transport | 0.01026902 |
| BP | icosanoid secretion | 0.01047172 |
| BP | toll-like receptor 4 signaling pathway | 0.01047172 |
| BP | necroptotic process | 0.01047172 |
| BP | vascular associated smooth muscle cell migration | 0.01047172 |
| BP | regulation of vascular associated smooth muscle cell migration | 0.01047172 |
| BP | regulation of transporter activity | 0.01053583 |
| BP | intrinsic apoptotic signaling pathway | 0.01053583 |
| BP | negative regulation of collagen metabolic process | 0.01062435 |
| BP | regulation of inositol phosphate biosynthetic process | 0.01062435 |
| BP | macrophage cytokine production | 0.01062435 |
| BP | skeletal muscle cell proliferation | 0.01062435 |
| BP | regulation of odontogenesis | 0.01062435 |
| BP | positive regulation of vascular permeability | 0.01062435 |
| BP | aldehyde biosynthetic process | 0.01062435 |
| BP | anatomical structure arrangement | 0.01062435 |
| BP | detection of temperature stimulus involved in sensory perception of pain | 0.01062435 |
| BP | phospholipid homeostasis | 0.01062435 |
| BP | hematopoietic stem cell homeostasis | 0.01062435 |
| BP | cellular response to prostaglandin E stimulus | 0.01062435 |
| BP | positive regulation of dendrite development | 0.01062435 |
| BP | regulation of NLRP3 inflammasome complex assembly | 0.01062435 |
| BP | negative regulation of lymphocyte migration | 0.01062435 |
| BP | positive regulation of neuron migration | 0.01062435 |
| BP | regulation of unsaturated fatty acid biosynthetic process | 0.01062435 |
| BP | central nervous system neuron development | 0.01063492 |
| BP | regulation of response to endoplasmic reticulum stress | 0.01063492 |
| BP | positive regulation of apoptotic signaling pathway | 0.01086255 |
| BP | defense response to bacterium | 0.01102429 |
| BP | metanephros development | 0.01109017 |
| BP | positive regulation of immunoglobulin production | 0.01115211 |
| BP | fibroblast migration | 0.01115211 |
| BP | positive regulation of receptor signaling pathway via JAK-STAT | 0.01115211 |
| BP | production of molecular mediator of immune response | 0.01117622 |
| BP | regulation of transforming growth factor beta receptor signaling pathway | 0.01121351 |
| BP | endoplasmic reticulum unfolded protein response | 0.01121351 |
| BP | positive regulation of proteolysis involved in cellular protein catabolic process | 0.01121351 |
| BP | regulation of cysteine-type endopeptidase activity | 0.01124787 |
| BP | negative regulation of proteolysis | 0.01141722 |
| BP | negative regulation of autophagy | 0.0115578 |
| BP | monocarboxylic acid transport | 0.01163723 |
| BP | endocrine pancreas development | 0.01185747 |
| BP | activin receptor signaling pathway | 0.01185747 |
| BP | negative regulation of toll-like receptor signaling pathway | 0.01185747 |
| BP | neuron maturation | 0.01185747 |
| BP | extrinsic apoptotic signaling pathway via death domain receptors | 0.01203793 |
| BP | regulation of Rho protein signal transduction | 0.01203793 |
| BP | regulation of cellular response to oxidative stress | 0.01203793 |
| BP | positive regulation of cellular extravasation | 0.01205849 |
| BP | positive regulation of type 2 immune response | 0.01205849 |
| BP | ovulation | 0.01205849 |
| BP | cellular response to hepatocyte growth factor stimulus | 0.01205849 |
| BP | NLRP3 inflammasome complex assembly | 0.01205849 |
| BP | regulation of MHC class II biosynthetic process | 0.01205849 |
| BP | hepatocyte growth factor receptor signaling pathway | 0.01205849 |
| BP | parasympathetic nervous system development | 0.01205849 |
| BP | coronary vasculature morphogenesis | 0.01205849 |
| BP | ganglion development | 0.01205849 |
| BP | glomerular mesangium development | 0.01205849 |
| BP | cellular response to thyroid hormone stimulus | 0.01205849 |
| BP | negative regulation of vascular endothelial growth factor signaling pathway | 0.01205849 |
| BP | vascular associated smooth muscle cell apoptotic process | 0.01205849 |
| BP | regulation of vascular associated smooth muscle cell apoptotic process | 0.01205849 |
| BP | response to organophosphorus | 0.01231268 |
| BP | regulation of cellular response to transforming growth factor beta stimulus | 0.01231268 |
| BP | adenylate cyclase-inhibiting G protein-coupled receptor signaling pathway | 0.01253067 |
| BP | positive regulation of protein localization to nucleus | 0.01253067 |
| BP | neuron recognition | 0.01258794 |
| BP | negative regulation of mitochondrion organization | 0.01258794 |
| BP | protein destabilization | 0.01258794 |
| BP | positive regulation of nucleotide metabolic process | 0.01258794 |
| BP | regulation of filopodium assembly | 0.01258794 |
| BP | cellular response to interleukin-6 | 0.01258794 |
| BP | vascular endothelial cell proliferation | 0.01258794 |
| BP | intermembrane lipid transfer | 0.01258794 |
| BP | positive regulation of purine nucleotide metabolic process | 0.01258794 |
| BP | regulation of vascular endothelial cell proliferation | 0.01258794 |
| BP | negative regulation of leukocyte activation | 0.01287439 |
| BP | negative regulation of transforming growth factor beta receptor signaling pathway | 0.01303613 |
| BP | positive regulation of cellular protein localization | 0.01327039 |
| BP | regulation of collagen metabolic process | 0.01334367 |
| BP | dopamine transport | 0.01334367 |
| BP | regulation of protein autophosphorylation | 0.01334367 |
| BP | negative regulation of type I interferon production | 0.01334367 |
| BP | negative regulation of cold-induced thermogenesis | 0.01334367 |
| BP | proteoglycan metabolic process | 0.01355443 |
| BP | energy reserve metabolic process | 0.01355443 |
| BP | membrane depolarization | 0.01355443 |
| BP | gastric acid secretion | 0.01357225 |
| BP | regulation of extracellular matrix disassembly | 0.01357225 |
| BP | intestinal cholesterol absorption | 0.01357225 |
| BP | sequestering of triglyceride | 0.01357225 |
| BP | response to caffeine | 0.01357225 |
| BP | regulation of integrin activation | 0.01357225 |
| BP | negative regulation of tyrosine phosphorylation of STAT protein | 0.01357225 |
| BP | MHC class II biosynthetic process | 0.01357225 |
| BP | detection of temperature stimulus involved in sensory perception | 0.01357225 |
| BP | negative regulation of cell division | 0.01357225 |
| BP | negative regulation of digestive system process | 0.01357225 |
| BP | chemokine (C-X-C motif) ligand 2 production | 0.01357225 |
| BP | positive regulation of mitochondrial fission | 0.01357225 |
| BP | negative regulation of release of cytochrome c from mitochondria | 0.01357225 |
| BP | dopamine uptake | 0.01357225 |
| BP | positive regulation of neuroinflammatory response | 0.01357225 |
| BP | regulation of chemokine (C-X-C motif) ligand 2 production | 0.01357225 |
| BP | response to unfolded protein | 0.01386086 |
| BP | Rho protein signal transduction | 0.01388889 |
| BP | positive regulation of proteasomal ubiquitin-dependent protein catabolic process | 0.01408565 |
| BP | positive regulation of T cell differentiation | 0.01408565 |
| BP | kidney morphogenesis | 0.01408565 |
| BP | nephron tubule development | 0.01408565 |
| BP | glandular epithelial cell differentiation | 0.01412476 |
| BP | negative regulation of myeloid leukocyte differentiation | 0.01412476 |
| BP | negative regulation of blood coagulation | 0.01412476 |
| BP | Notch signaling pathway | 0.01420109 |
| BP | regulation of plasma membrane bounded cell projection assembly | 0.01420109 |
| BP | placenta development | 0.01430316 |
| BP | positive regulation of cysteine-type endopeptidase activity involved in apoptotic process | 0.01430316 |
| BP | regulation of stress fiber assembly | 0.01462992 |
| BP | neutral lipid metabolic process | 0.01472567 |
| BP | acylglycerol metabolic process | 0.01472567 |
| BP | actin filament polymerization | 0.01489897 |
| BP | regulation of cell projection assembly | 0.01489897 |
| BP | regulation of chondrocyte differentiation | 0.01493132 |
| BP | positive regulation of alpha-beta T cell differentiation | 0.01493132 |
| BP | mesenchyme morphogenesis | 0.01493132 |
| BP | negative regulation of hemostasis | 0.01493132 |
| BP | positive regulation of protein modification by small protein conjugation or removal | 0.01515647 |
| BP | natural killer cell differentiation | 0.0151638 |
| BP | regulation of systemic arterial blood pressure by circulatory renin-angiotensin | 0.0151638 |
| BP | complement activation, alternative pathway | 0.0151638 |
| BP | blood coagulation, intrinsic pathway | 0.0151638 |
| BP | hyaluronan catabolic process | 0.0151638 |
| BP | negative regulation of nervous system process | 0.0151638 |
| BP | response to magnesium ion | 0.0151638 |
| BP | regulation of interleukin-13 production | 0.0151638 |
| BP | negative regulation of monooxygenase activity | 0.0151638 |
| BP | response to vitamin A | 0.0151638 |
| BP | high-density lipoprotein particle remodeling | 0.0151638 |
| BP | response to hepatocyte growth factor | 0.0151638 |
| BP | negative regulation of nitric oxide biosynthetic process | 0.0151638 |
| BP | negative regulation of gluconeogenesis | 0.0151638 |
| BP | keratinocyte migration | 0.0151638 |
| BP | negative regulation of focal adhesion assembly | 0.0151638 |
| BP | positive regulation of glial cell proliferation | 0.0151638 |
| BP | negative regulation of necroptotic process | 0.0151638 |
| BP | catecholamine uptake | 0.0151638 |
| BP | negative regulation of cell-substrate junction organization | 0.0151638 |
| BP | negative regulation of cellular response to vascular endothelial growth factor stimulus | 0.0151638 |
| BP | negative regulation of nitric oxide metabolic process | 0.0151638 |
| BP | bone morphogenesis | 0.01518732 |
| BP | renal tubule development | 0.01518732 |
| BP | negative regulation of response to wounding | 0.01518732 |
| BP | cellular response to metal ion | 0.01525671 |
| BP | pattern specification process | 0.01527795 |
| BP | regulation of heart contraction | 0.01545402 |
| BP | steroid biosynthetic process | 0.01562036 |
| BP | regulation of Ras protein signal transduction | 0.01562036 |
| BP | positive regulation of viral process | 0.01575795 |
| BP | telencephalon cell migration | 0.01576345 |
| BP | regulation of interferon-beta production | 0.01576345 |
| BP | negative regulation of osteoblast differentiation | 0.01576345 |
| BP | negative regulation of G protein-coupled receptor signaling pathway | 0.01576345 |
| BP | response to interleukin-6 | 0.01576345 |
| BP | actomyosin structure organization | 0.01598999 |
| BP | negative regulation of protein-containing complex assembly | 0.0160432 |
| BP | regulation of metal ion transport | 0.01608914 |
| BP | negative regulation of BMP signaling pathway | 0.01662121 |
| BP | collagen biosynthetic process | 0.01662121 |
| BP | icosanoid transport | 0.01662121 |
| BP | programmed necrotic cell death | 0.01662121 |
| BP | homeostasis of number of cells | 0.01674259 |
| BP | blastocyst growth | 0.01683136 |
| BP | morphogenesis of an endothelium | 0.01683136 |
| BP | negative regulation of adenylate cyclase activity | 0.01683136 |
| BP | axonal fasciculation | 0.01683136 |
| BP | retinal ganglion cell axon guidance | 0.01683136 |
| BP | regulation of response to food | 0.01683136 |
| BP | interleukin-5 production | 0.01683136 |
| BP | regulation of interleukin-5 production | 0.01683136 |
| BP | positive regulation of mast cell activation | 0.01683136 |
| BP | toll-like receptor 3 signaling pathway | 0.01683136 |
| BP | wound healing, spreading of epidermal cells | 0.01683136 |
| BP | negative regulation of macrophage activation | 0.01683136 |
| BP | regulation of mitochondrial depolarization | 0.01683136 |
| BP | negative regulation of cardiac muscle cell proliferation | 0.01683136 |
| BP | definitive hemopoiesis | 0.01683136 |
| BP | endothelial tube morphogenesis | 0.01683136 |
| BP | negative regulation of programmed necrotic cell death | 0.01683136 |
| BP | intestinal lipid absorption | 0.01683136 |
| BP | neuron projection fasciculation | 0.01683136 |
| BP | regulation of glial cell migration | 0.01683136 |
| BP | regulation of gliogenesis | 0.01693927 |
| BP | regulation of response to oxidative stress | 0.01693927 |
| BP | positive regulation of cation transmembrane transport | 0.01696389 |
| BP | interferon-beta production | 0.01750468 |
| BP | negative regulation of coagulation | 0.01750468 |
| BP | regulation of nitric-oxide synthase activity | 0.01750468 |
| BP | endochondral bone morphogenesis | 0.01750468 |
| BP | plasma lipoprotein particle organization | 0.01750468 |
| BP | phospholipid transport | 0.01755014 |
| BP | primary alcohol metabolic process | 0.01755014 |
| BP | positive regulation of neuron differentiation | 0.01755014 |
| BP | protein import into nucleus | 0.01791901 |
| BP | insulin receptor signaling pathway | 0.01791901 |
| BP | response to starvation | 0.01792918 |
| BP | regulation of G protein-coupled receptor signaling pathway | 0.01840963 |
| BP | positive regulation of calcium ion transport into cytosol | 0.01841392 |
| BP | inositol phosphate-mediated signaling | 0.01841392 |
| BP | serotonin transport | 0.01857316 |
| BP | inflammatory cell apoptotic process | 0.01857316 |
| BP | male genitalia development | 0.01857316 |
| BP | interleukin-13 production | 0.01857316 |
| BP | negative regulation of organic acid transport | 0.01857316 |
| BP | reverse cholesterol transport | 0.01857316 |
| BP | negative regulation of nucleocytoplasmic transport | 0.01857316 |
| BP | response to corticosterone | 0.01857316 |
| BP | labyrinthine layer morphogenesis | 0.01857316 |
| BP | positive regulation of monocyte chemotaxis | 0.01857316 |
| BP | positive regulation of calcium ion import | 0.01857316 |
| BP | negative regulation of hydrogen peroxide-induced cell death | 0.01857316 |
| BP | regulation of microglial cell activation | 0.01857316 |
| BP | negative regulation of cellular catabolic process | 0.01881547 |
| BP | cellular glucose homeostasis | 0.01890905 |
| BP | neuron migration | 0.01890905 |
| BP | myeloid cell homeostasis | 0.01890905 |
| BP | retina development in camera-type eye | 0.01890905 |
| BP | sodium ion homeostasis | 0.01934896 |
| BP | positive regulation of peptidase activity | 0.01959238 |
| BP | skeletal muscle tissue development | 0.01993446 |
| BP | regulation of actomyosin structure organization | 0.02013021 |
| BP | regulation of small GTPase mediated signal transduction | 0.02024523 |
| BP | release of cytochrome c from mitochondria | 0.02030985 |
| BP | ruffle organization | 0.02030985 |
| BP | organ induction | 0.02038747 |
| BP | mating behavior | 0.02038747 |
| BP | peroxisome proliferator activated receptor signaling pathway | 0.02038747 |
| BP | lipid digestion | 0.02038747 |
| BP | negative regulation of gene silencing by miRNA | 0.02038747 |
| BP | hepatocyte proliferation | 0.02038747 |
| BP | epithelial cell proliferation involved in liver morphogenesis | 0.02038747 |
| BP | regulation of cAMP-dependent protein kinase activity | 0.02038747 |
| BP | regulation of ATP biosynthetic process | 0.02038747 |
| BP | response to radiation | 0.02059937 |
| BP | positive regulation of supramolecular fiber organization | 0.02090678 |
| BP | cellular response to unfolded protein | 0.0209957 |
| BP | alpha-beta T cell activation | 0.0209957 |
| BP | positive regulation of interleukin-1 beta production | 0.02129661 |
| BP | protein-lipid complex subunit organization | 0.02129661 |
| BP | CD4-positive, alpha-beta T cell activation | 0.02150326 |
| BP | positive regulation of cysteine-type endopeptidase activity | 0.02209318 |
| BP | positive regulation of lymphocyte differentiation | 0.02221078 |
| BP | regulation of systemic arterial blood pressure by renin-angiotensin | 0.02227257 |
| BP | phasic smooth muscle contraction | 0.02227257 |
| BP | maintenance of gastrointestinal epithelium | 0.02227257 |
| BP | positive regulation of nucleotide biosynthetic process | 0.02227257 |
| BP | negative regulation of cyclase activity | 0.02227257 |
| BP | negative regulation of muscle contraction | 0.02227257 |
| BP | negative regulation of phagocytosis | 0.02227257 |
| BP | mitochondrial depolarization | 0.02227257 |
| BP | liver morphogenesis | 0.02227257 |
| BP | positive regulation of purine nucleotide biosynthetic process | 0.02227257 |
| BP | negative regulation of purine nucleotide metabolic process | 0.02227257 |
| BP | negative regulation of response to reactive oxygen species | 0.02227257 |
| BP | regulation of oxidative stress-induced neuron death | 0.02227257 |
| BP | positive regulation of blood vessel endothelial cell proliferation involved in sprouting angiogenesis | 0.02227257 |
| BP | response to topologically incorrect protein | 0.02227978 |
| BP | negative regulation of intracellular transport | 0.02230925 |
| BP | positive regulation of Notch signaling pathway | 0.02230925 |
| BP | phosphatidylinositol phosphorylation | 0.02230925 |
| BP | calcium ion transport into cytosol | 0.02265564 |
| BP | positive regulation of myeloid cell differentiation | 0.02293238 |
| BP | leukocyte apoptotic process | 0.02293238 |
| BP | male sex differentiation | 0.02322731 |
| BP | morphogenesis of an epithelial sheet | 0.02334778 |
| BP | regulation of vascular endothelial growth factor production | 0.02334778 |
| BP | neuron projection regeneration | 0.02334778 |
| BP | regulation of autophagy | 0.02343339 |
| BP | interleukin-1-mediated signaling pathway | 0.02366812 |
| BP | negative regulation of extrinsic apoptotic signaling pathway | 0.02366812 |
| BP | cellular response to nutrient levels | 0.02420338 |
| BP | acute inflammatory response to antigenic stimulus | 0.02422678 |
| BP | regulation of protein kinase A signaling | 0.02422678 |
| BP | calcium-independent cell-cell adhesion via plasma membrane cell-adhesion molecules | 0.02422678 |
| BP | positive regulation of blood coagulation | 0.02422678 |
| BP | regulation of myeloid cell apoptotic process | 0.02422678 |
| BP | embryonic hemopoiesis | 0.02422678 |
| BP | PERK-mediated unfolded protein response | 0.02422678 |
| BP | positive regulation of fatty acid biosynthetic process | 0.02422678 |
| BP | negative regulation of nucleotide metabolic process | 0.02422678 |
| BP | sensory perception of temperature stimulus | 0.02422678 |
| BP | negative regulation of posttranscriptional gene silencing | 0.02422678 |
| BP | negative regulation of necrotic cell death | 0.02422678 |
| BP | negative regulation of gene silencing by RNA | 0.02422678 |
| BP | response to thyroid hormone | 0.02422678 |
| BP | amelogenesis | 0.02422678 |
| BP | positive regulation of hemostasis | 0.02422678 |
| BP | positive regulation of extracellular matrix organization | 0.02422678 |
| BP | positive regulation of vascular associated smooth muscle cell migration | 0.02422678 |
| BP | positive regulation of endothelial cell apoptotic process | 0.02422678 |
| BP | contractile actin filament bundle assembly | 0.02441805 |
| BP | stress fiber assembly | 0.02441805 |
| BP | positive regulation of ubiquitin-dependent protein catabolic process | 0.02441805 |
| BP | gonad development | 0.02520566 |
| BP | negative regulation of Wnt signaling pathway | 0.02520566 |
| BP | positive regulation of macroautophagy | 0.02550245 |
| BP | positive regulation of plasma membrane bounded cell projection assembly | 0.02596071 |
| BP | regulation of NIK/NF-kappaB signaling | 0.02596071 |
| BP | cell-cell adhesion via plasma-membrane adhesion molecules | 0.02610393 |
| BP | endocardial cushion formation | 0.02624846 |
| BP | substrate-dependent cell migration | 0.02624846 |
| BP | negative regulation of tumor necrosis factor-mediated signaling pathway | 0.02624846 |
| BP | regulation of vesicle fusion | 0.02624846 |
| BP | response to gonadotropin | 0.02624846 |
| BP | negative regulation of fibroblast growth factor receptor signaling pathway | 0.02624846 |
| BP | regulation of insulin-like growth factor receptor signaling pathway | 0.02624846 |
| BP | regulation of astrocyte differentiation | 0.02624846 |
| BP | positive regulation of coagulation | 0.02624846 |
| BP | myoblast proliferation | 0.02624846 |
| BP | embryonic placenta morphogenesis | 0.02624846 |
| BP | detection of stimulus involved in sensory perception of pain | 0.02624846 |
| BP | positive regulation of macrophage migration | 0.02624846 |
| BP | anatomical structure homeostasis | 0.02648334 |
| BP | positive regulation of epithelial cell differentiation | 0.02661856 |
| BP | response to cadmium ion | 0.02661856 |
| BP | positive regulation of nucleocytoplasmic transport | 0.02661856 |
| BP | memory | 0.02675354 |
| BP | positive regulation of NF-kappaB transcription factor activity | 0.02685339 |
| BP | regulation of muscle cell differentiation | 0.02685339 |
| BP | cellular response to inorganic substance | 0.02729257 |
| BP | substrate adhesion-dependent cell spreading | 0.02756075 |
| BP | response to monoamine | 0.02756075 |
| BP | response to catecholamine | 0.02756075 |
| BP | inflammatory response to antigenic stimulus | 0.02776047 |
| BP | vascular endothelial growth factor production | 0.02776047 |
| BP | proteoglycan biosynthetic process | 0.02776047 |
| BP | platelet aggregation | 0.02776047 |
| BP | development of primary sexual characteristics | 0.02783164 |
| BP | regulation of muscle contraction | 0.02813781 |
| BP | negative regulation of supramolecular fiber organization | 0.02813781 |
| BP | positive regulation of receptor internalization | 0.02833598 |
| BP | forebrain neuron development | 0.02833598 |
| BP | response to immobilization stress | 0.02833598 |
| BP | fibrinolysis | 0.02833598 |
| BP | astrocyte activation | 0.02833598 |
| BP | negative regulation of lyase activity | 0.02833598 |
| BP | killing by host of symbiont cells | 0.02833598 |
| BP | regulation of feeding behavior | 0.02833598 |
| BP | regulation of response to interferon-gamma | 0.02833598 |
| BP | regulation of interferon-gamma-mediated signaling pathway | 0.02833598 |
| BP | regulation of necroptotic process | 0.02833598 |
| BP | secondary palate development | 0.02833598 |
| BP | negative regulation of vascular endothelial cell proliferation | 0.02833598 |
| BP | heart contraction | 0.0289105 |
| BP | regulation of osteoclast differentiation | 0.02892814 |
| BP | filopodium assembly | 0.02892814 |
| BP | negative regulation of reactive oxygen species metabolic process | 0.02892814 |
| BP | regulation of oxidoreductase activity | 0.0292185 |
| BP | import into nucleus | 0.02946064 |
| BP | calcium ion transport | 0.02994569 |
| BP | skeletal muscle cell differentiation | 0.03012152 |
| BP | T-helper cell differentiation | 0.03012152 |
| BP | inactivation of MAPK activity | 0.03048773 |
| BP | regulation of animal organ formation | 0.03048773 |
| BP | cranial nerve morphogenesis | 0.03048773 |
| BP | olfactory bulb development | 0.03048773 |
| BP | central nervous system projection neuron axonogenesis | 0.03048773 |
| BP | regulation of response to extracellular stimulus | 0.03048773 |
| BP | regulation of response to nutrient levels | 0.03048773 |
| BP | regulation of toll-like receptor 4 signaling pathway | 0.03048773 |
| BP | neuron death in response to oxidative stress | 0.03048773 |
| BP | positive regulation of activated T cell proliferation | 0.03048773 |
| BP | regulation of SMAD protein signal transduction | 0.03048773 |
| BP | regulation of protein insertion into mitochondrial membrane involved in apoptotic signaling pathway | 0.03048773 |
| BP | positive regulation of protein insertion into mitochondrial membrane involved in apoptotic signaling pathway | 0.03048773 |
| BP | regulation of vascular endothelial growth factor signaling pathway | 0.03048773 |
| BP | regulation of lymphocyte chemotaxis | 0.03048773 |
| BP | regulation of hydrogen peroxide-induced cell death | 0.03048773 |
| BP | glial cell development | 0.03093427 |
| BP | release of sequestered calcium ion into cytosol | 0.03093427 |
| BP | embryonic organ development | 0.03116739 |
| BP | embryonic pattern specification | 0.03134055 |
| BP | plasma lipoprotein particle clearance | 0.03134055 |
| BP | necrotic cell death | 0.03134055 |
| BP | positive regulation of DNA biosynthetic process | 0.03134055 |
| BP | carboxylic acid transport | 0.03140022 |
| BP | leukocyte mediated cytotoxicity | 0.03181399 |
| BP | negative regulation of sequestering of calcium ion | 0.03181399 |
| BP | CD4-positive, alpha-beta T cell differentiation involved in immune response | 0.03258517 |
| BP | positive regulation of alpha-beta T cell activation | 0.03258517 |
| BP | negative regulation of signaling receptor activity | 0.03258517 |
| BP | regulation of intracellular transport | 0.03268255 |
| BP | regulation of T-helper 1 type immune response | 0.03270216 |
| BP | positive regulation of toll-like receptor signaling pathway | 0.03270216 |
| BP | embryonic hindlimb morphogenesis | 0.03270216 |
| BP | negative regulation of osteoclast differentiation | 0.03270216 |
| BP | response to pain | 0.03270216 |
| BP | killing of cells in other organism involved in symbiotic interaction | 0.03270216 |
| BP | integrated stress response signaling | 0.03270216 |
| BP | negative regulation of response to oxidative stress | 0.03270216 |
| BP | cellular response to topologically incorrect protein | 0.03293761 |
| BP | regulation of sequestering of calcium ion | 0.03361722 |
| BP | muscle contraction | 0.03363119 |
| BP | alpha-beta T cell activation involved in immune response | 0.0338553 |
| BP | alpha-beta T cell differentiation involved in immune response | 0.0338553 |
| BP | regulation of immunoglobulin production | 0.0338553 |
| BP | G protein-coupled receptor signaling pathway, coupled to cyclic nucleotide second messenger | 0.0338553 |
| BP | transmission of nerve impulse | 0.0338553 |
| BP | protein localization to cell surface | 0.0338553 |
| BP | regulation of alpha-beta T cell differentiation | 0.0338553 |
| BP | regulation of calcium-mediated signaling | 0.0338553 |
| BP | positive regulation of NIK/NF-kappaB signaling | 0.0338553 |
| BP | positive regulation of protein localization to cell periphery | 0.0338553 |
| BP | heart process | 0.03403024 |
| BP | endochondral ossification | 0.0349777 |
| BP | olfactory lobe development | 0.0349777 |
| BP | replacement ossification | 0.0349777 |
| BP | regulation of programmed necrotic cell death | 0.0349777 |
| BP | protein activation cascade | 0.0349777 |
| BP | blood coagulation, fibrin clot formation | 0.0349777 |
| BP | neuron projection arborization | 0.0349777 |
| BP | regulation of cellular response to vascular endothelial growth factor stimulus | 0.0349777 |
| BP | positive regulation of sprouting angiogenesis | 0.0349777 |
| BP | regulation of glial cell differentiation | 0.03515086 |
| BP | regulation of oxidative stress-induced cell death | 0.03515086 |
| BP | adult behavior | 0.03547901 |
| BP | cellular response to oxygen levels | 0.03612711 |
| BP | regulation of circadian rhythm | 0.03643191 |
| BP | regulation of DNA binding | 0.03643191 |
| BP | cardiac ventricle morphogenesis | 0.03647175 |
| BP | regulation of insulin receptor signaling pathway | 0.03647175 |
| BP | cellular response to acid chemical | 0.03647175 |
| BP | positive regulation of membrane permeability | 0.03647175 |
| BP | cytosolic calcium ion transport | 0.03666045 |
| BP | cell recognition | 0.03677418 |
| BP | protein polymerization | 0.03680324 |
| BP | cell junction assembly | 0.03686411 |
| BP | cardiac atrium morphogenesis | 0.03731283 |
| BP | epithelial structure maintenance | 0.03731283 |
| BP | positive regulation of multicellular organism growth | 0.03731283 |
| BP | positive regulation of macrophage activation | 0.03731283 |
| BP | arachidonic acid secretion | 0.03731283 |
| BP | response to cholesterol | 0.03731283 |
| BP | regulation of ruffle assembly | 0.03731283 |
| BP | regulation of endoplasmic reticulum unfolded protein response | 0.03731283 |
| BP | positive regulation of p38MAPK cascade | 0.03731283 |
| BP | arachidonate transport | 0.03731283 |
| BP | response to acid chemical | 0.0373995 |
| BP | sequestering of calcium ion | 0.0373995 |
| BP | regulation of ATP metabolic process | 0.0373995 |
| BP | artery morphogenesis | 0.03781789 |
| BP | regulation of lymphocyte differentiation | 0.0382192 |
| BP | regulation of animal organ morphogenesis | 0.03901358 |
| BP | diencephalon development | 0.03918917 |
| BP | positive regulation of insulin secretion | 0.03918917 |
| BP | inositol phosphate metabolic process | 0.03918917 |
| BP | regulation of protein localization to cell periphery | 0.03937876 |
| BP | protein insertion into mitochondrial membrane involved in apoptotic signaling pathway | 0.03970607 |
| BP | response to amphetamine | 0.03970607 |
| BP | positive regulation of interleukin-2 production | 0.03970607 |
| BP | negative regulation of peptidyl-serine phosphorylation | 0.03970607 |
| BP | cell death in response to hydrogen peroxide | 0.03970607 |
| BP | eating behavior | 0.03970607 |
| BP | regulation of B cell differentiation | 0.03970607 |
| BP | positive regulation of synaptic transmission, glutamatergic | 0.03970607 |
| BP | placenta blood vessel development | 0.03970607 |
| BP | interleukin-6-mediated signaling pathway | 0.03970607 |
| BP | cellular response to vitamin | 0.03970607 |
| BP | basement membrane organization | 0.03970607 |
| BP | negative regulation of protein localization to nucleus | 0.03970607 |
| BP | negative regulation of intrinsic apoptotic signaling pathway in response to DNA damage | 0.03970607 |
| BP | regulation of cation channel activity | 0.039818 |
| BP | regulation of protein modification by small protein conjugation or removal | 0.04012099 |
| BP | lamellipodium assembly | 0.04058548 |
| BP | cell adhesion mediated by integrin | 0.04058548 |
| BP | negative regulation of cysteine-type endopeptidase activity involved in apoptotic process | 0.04058548 |
| BP | lipid phosphorylation | 0.04058548 |
| BP | central nervous system neuron differentiation | 0.04063247 |
| BP | regulation of calcium ion transport | 0.04081278 |
| BP | mesoderm development | 0.04141686 |
| BP | negative regulation of canonical Wnt signaling pathway | 0.04145702 |
| BP | positive regulation of intracellular protein transport | 0.04145702 |
| CC | collagen-containing extracellular matrix | 1.17E-11 |
| CC | external side of plasma membrane | 3.54E-08 |
| CC | platelet alpha granule | 4.34E-06 |
| CC | endocytic vesicle | 1.78E-05 |
| CC | membrane raft | 3.03E-05 |
| CC | membrane microdomain | 3.03E-05 |
| CC | plasma lipoprotein particle | 3.13E-05 |
| CC | lipoprotein particle | 3.13E-05 |
| CC | protein-lipid complex | 4.66E-05 |
| CC | platelet alpha granule lumen | 6.37E-05 |
| CC | high-density lipoprotein particle | 0.00015602 |
| CC | transcription regulator complex | 0.00031964 |
| CC | microvillus | 0.00034722 |
| CC | secretory granule lumen | 0.00050806 |
| CC | cytoplasmic vesicle lumen | 0.00056296 |
| CC | vesicle lumen | 0.00059222 |
| CC | caveola | 0.00138601 |
| CC | early endosome | 0.00185612 |
| CC | RNA polymerase II transcription regulator complex | 0.00188195 |
| CC | phosphatidylinositol 3-kinase complex | 0.00318163 |
| CC | blood microparticle | 0.00397627 |
| MF | signaling receptor activator activity | 8.84E-30 |
| MF | receptor ligand activity | 6.11E-29 |
| MF | growth factor binding | 2.28E-18 |
| MF | cytokine binding | 3.52E-17 |
| MF | cytokine activity | 1.17E-16 |
| MF | cytokine receptor binding | 2.70E-16 |
| MF | glycosaminoglycan binding | 6.92E-16 |
| MF | growth factor activity | 1.16E-15 |
| MF | chemokine activity | 4.15E-15 |
| MF | transmembrane receptor protein kinase activity | 1.83E-13 |
| MF | heparin binding | 2.61E-13 |
| MF | chemokine receptor binding | 5.04E-13 |
| MF | G protein-coupled receptor binding | 8.28E-12 |
| MF | sulfur compound binding | 9.46E-12 |
| MF | hormone binding | 1.23E-10 |
| MF | peptide hormone binding | 1.40E-10 |
| MF | nuclear receptor activity | 2.12E-10 |
| MF | ligand-activated transcription factor activity | 2.12E-10 |
| MF | peptide binding | 2.97E-10 |
| MF | CCR chemokine receptor binding | 1.48E-09 |
| MF | amide binding | 2.18E-09 |
| MF | growth factor receptor binding | 5.17E-09 |
| MF | G protein-coupled peptide receptor activity | 9.34E-09 |
| MF | peptide receptor activity | 1.29E-08 |
| MF | transmembrane receptor protein tyrosine kinase activity | 2.02E-08 |
| MF | cytokine receptor activity | 1.10E-07 |
| MF | integrin binding | 4.76E-07 |
| MF | chemokine binding | 1.39E-06 |
| MF | transmembrane receptor protein serine/threonine kinase activity | 1.55E-06 |
| MF | protein tyrosine kinase activity | 2.40E-06 |
| MF | immune receptor activity | 2.57E-06 |
| MF | prostaglandin receptor activity | 2.79E-06 |
| MF | prostanoid receptor activity | 4.35E-06 |
| MF | transforming growth factor beta binding | 5.40E-06 |
| MF | fibroblast growth factor receptor binding | 6.69E-06 |
| MF | steroid hormone receptor activity | 8.21E-06 |
| MF | transforming growth factor beta-activated receptor activity | 9.27E-06 |
| MF | low-density lipoprotein particle receptor activity | 1.29E-05 |
| MF | icosanoid receptor activity | 1.74E-05 |
| MF | cargo receptor activity | 1.81E-05 |
| MF | hormone receptor binding | 2.16E-05 |
| MF | lipoprotein particle receptor activity | 2.98E-05 |
| MF | CXCR chemokine receptor binding | 3.80E-05 |
| MF | chemoattractant activity | 4.93E-05 |
| MF | semaphorin receptor binding | 0.00010537 |
| MF | steroid binding | 0.00011591 |
| MF | C-C chemokine binding | 0.00012537 |
| MF | DNA-binding transcription activator activity | 0.00012777 |
| MF | activin-activated receptor activity | 0.00014712 |
| MF | scavenger receptor activity | 0.00015889 |
| MF | G protein-coupled chemoattractant receptor activity | 0.00017338 |
| MF | chemokine receptor activity | 0.00017338 |
| MF | coreceptor activity | 0.0001758 |
| MF | cell-cell adhesion mediator activity | 0.00019405 |
| MF | SMAD binding | 0.00019992 |
| MF | chemorepellent activity | 0.0002018 |
| MF | receptor serine/threonine kinase binding | 0.00023342 |
| MF | glucocorticoid receptor binding | 0.00026538 |
| MF | lipoprotein particle binding | 0.00030712 |
| MF | protein-lipid complex binding | 0.00030712 |
| MF | enzyme inhibitor activity | 0.00033997 |
| MF | BMP receptor binding | 0.00034221 |
| MF | alcohol binding | 0.00036542 |
| MF | DNA-binding transcription activator activity, RNA polymerase II-specific | 0.00039387 |
| MF | hormone activity | 0.00039623 |
| MF | cell adhesion mediator activity | 0.00043058 |
| MF | S100 protein binding | 0.00043203 |
| MF | platelet-derived growth factor receptor binding | 0.00053569 |
| MF | activin binding | 0.00053569 |
| MF | proteoglycan binding | 0.00062711 |
| MF | insulin-like growth factor receptor binding | 0.000654 |
| MF | 1-phosphatidylinositol-3-kinase regulator activity | 0.000654 |
| MF | low-density lipoprotein particle binding | 0.00078775 |
| MF | apolipoprotein binding | 0.00078775 |
| MF | heparan sulfate proteoglycan binding | 0.00078775 |
| MF | phosphatidylinositol 3-kinase regulator activity | 0.00128905 |
| MF | cholesterol transfer activity | 0.00149182 |
| MF | steroid hormone receptor binding | 0.00157007 |
| MF | sterol transfer activity | 0.00171351 |
| MF | C-C chemokine receptor activity | 0.00195473 |
| MF | lipid transporter activity | 0.00208965 |
| MF | cholesterol binding | 0.00217602 |
| MF | transmembrane receptor protein serine/threonine kinase binding | 0.00221606 |
| MF | amyloid-beta binding | 0.00230679 |
| MF | calcium-dependent protein binding | 0.00230679 |
| MF | phospholipid transporter activity | 0.00269715 |
| MF | extracellular matrix binding | 0.00308849 |
| MF | RNA polymerase II-specific DNA-binding transcription factor binding | 0.00309242 |
| MF | insulin-like growth factor binding | 0.00384317 |
| MF | sterol binding | 0.00423257 |
| MF | nuclear receptor binding | 0.00429652 |
| MF | nuclear hormone receptor binding | 0.00448558 |
| MF | phosphatidylcholine binding | 0.00465247 |
| MF | quaternary ammonium group binding | 0.00465247 |
| MF | phosphatidylinositol 3-kinase regulatory subunit binding | 0.00507753 |
| MF | phosphatidylinositol 3-kinase binding | 0.00509278 |
| MF | sterol transporter activity | 0.00604657 |
| MF | mitogen-activated protein kinase kinase binding | 0.00616128 |
| MF | protein binding involved in heterotypic cell-cell adhesion | 0.00616128 |
| MF | RNA polymerase II repressing transcription factor binding | 0.00656073 |
| MF | protein kinase inhibitor activity | 0.00695373 |
| MF | signaling adaptor activity | 0.00731317 |
| MF | neurotrophin receptor binding | 0.00734046 |
| MF | high-density lipoprotein particle binding | 0.00734046 |
| MF | semaphorin receptor activity | 0.00734046 |
| MF | kinase inhibitor activity | 0.0084644 |
| MF | ubiquitin-ubiquitin ligase activity | 0.00861287 |
| MF | long-chain fatty acid binding | 0.00861287 |
| MF | DNA-binding transcription factor binding | 0.00872299 |
| MF | vascular endothelial growth factor receptor binding | 0.00997634 |
| MF | kinase regulator activity | 0.01088428 |
| MF | extracellular matrix structural constituent | 0.01121794 |
| MF | retinol binding | 0.012968 |
| MF | cAMP response element binding | 0.012968 |
| MF | peptidase regulator activity | 0.01363162 |
| MF | lipid transfer activity | 0.01393844 |
| MF | endopeptidase inhibitor activity | 0.01453616 |
| MF | long-chain fatty acid transporter activity | 0.01459203 |
| MF | phosphatidylcholine transporter activity | 0.01459203 |
| MF | retinal binding | 0.01629882 |
| MF | mitogen-activated protein kinase kinase kinase binding | 0.01629882 |
| MF | sphingolipid transporter activity | 0.01629882 |
| MF | peptidase inhibitor activity | 0.01723031 |
