# Supplementary figures and images for: Development of a Novel Immune-Related Gene Prognostic Index for Breast Cancer
Source: Front Immunol. 2022 Apr 26;13:845093. doi: 10.3389/fimmu.2022.845093 (PMC9086776; doi:10.3389/fimmu.2022.845093)

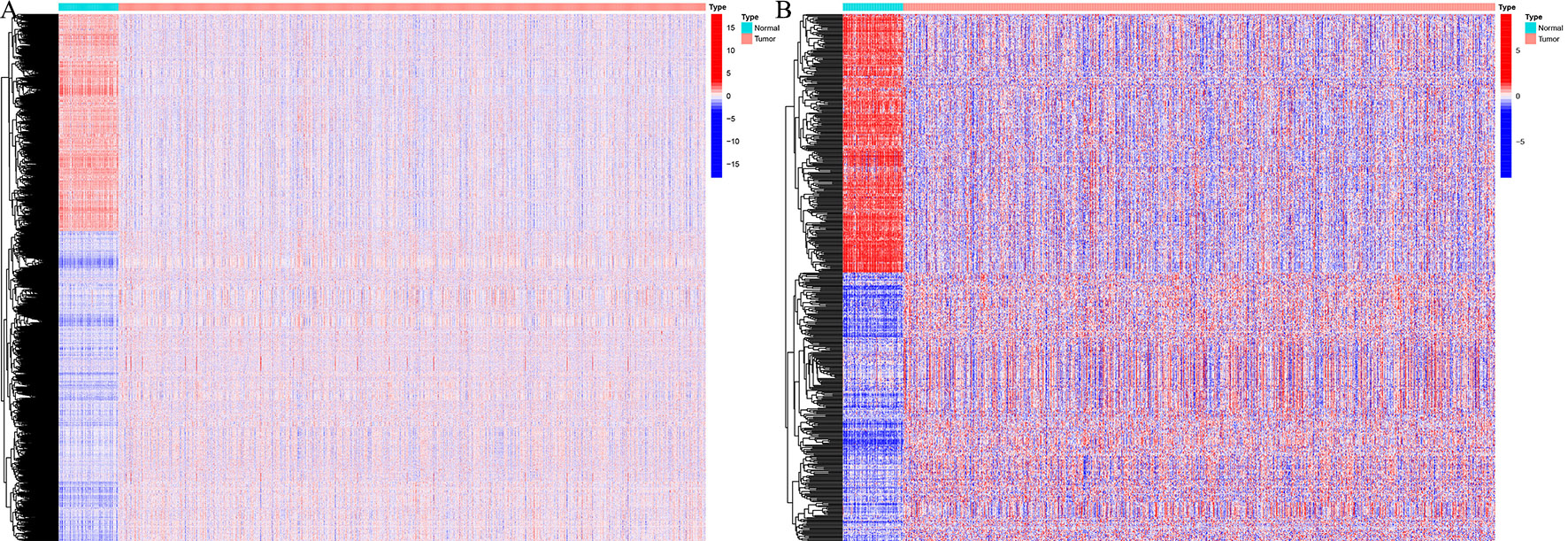

Supplement: Supplementary file 1 [file DataSheet_1.zip › Data Sheet 1 (1)/Supplementary/Supplementary Figure 1.jpg]

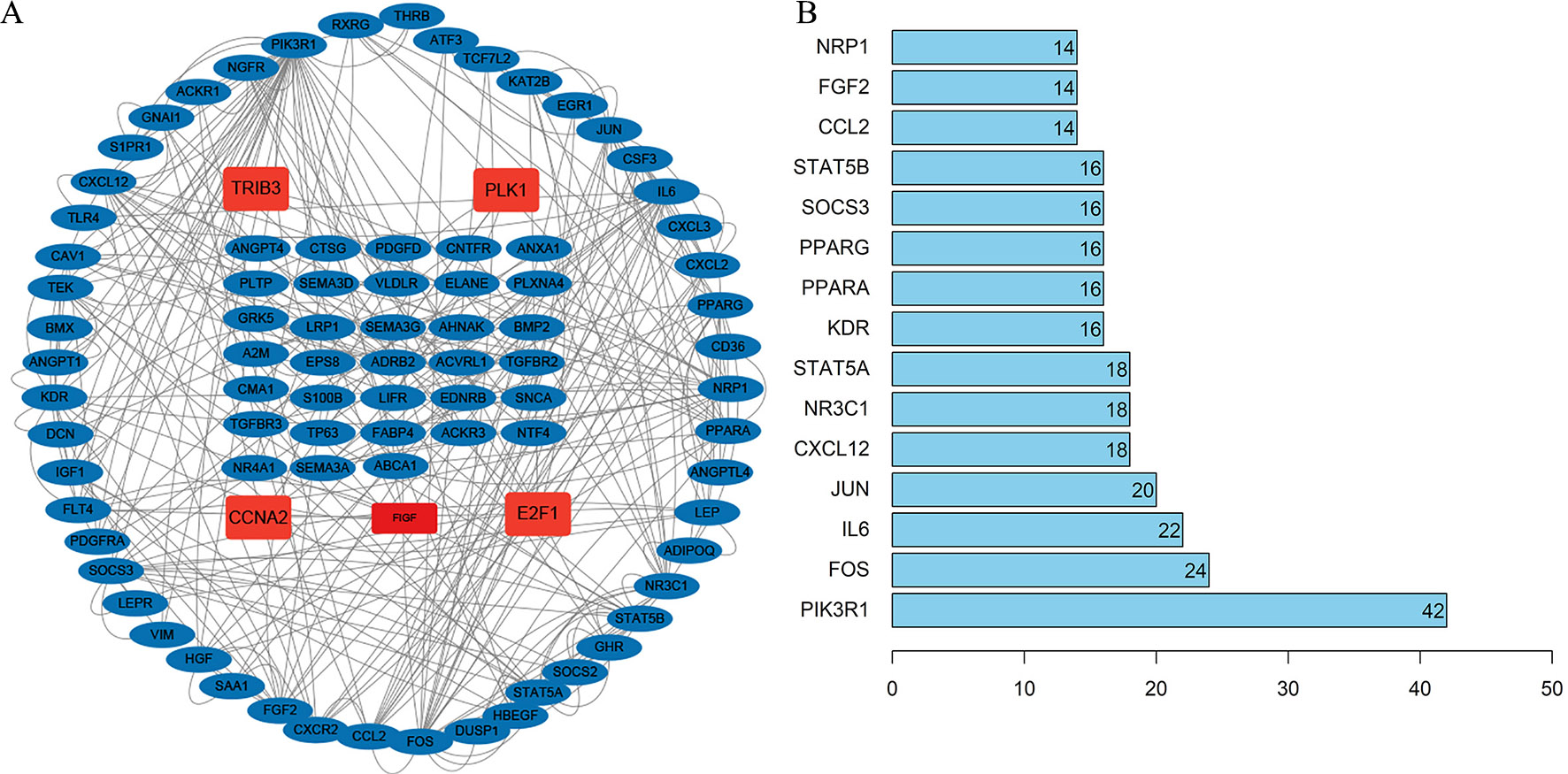

Supplement: Supplementary file 1 [file DataSheet_1.zip › Data Sheet 1 (1)/Supplementary/Supplementary Figure 2.jpg]

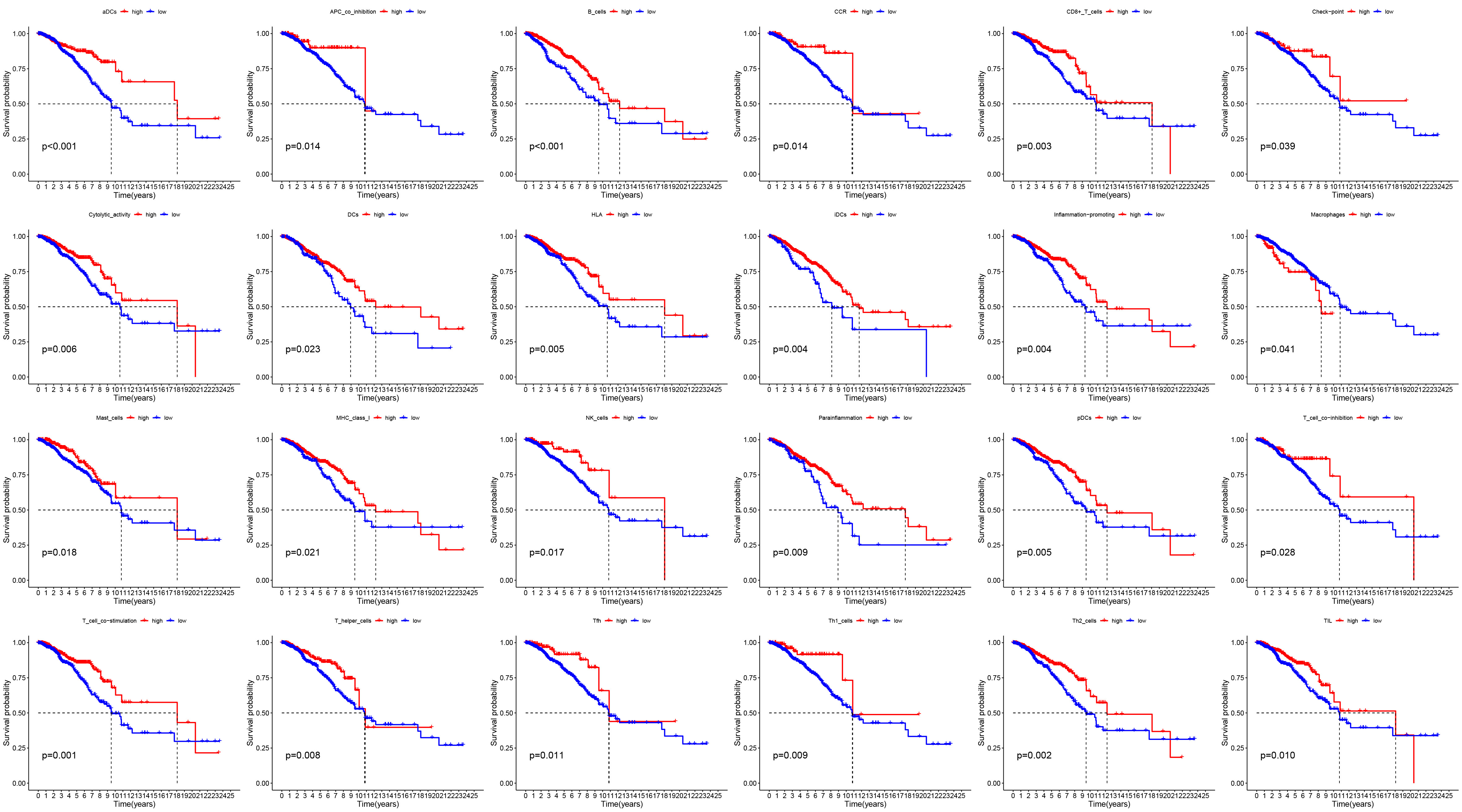

Supplement: Supplementary file 1 [file DataSheet_1.zip › Data Sheet 1 (1)/Supplementary/Supplementary Figure 4.jpg]

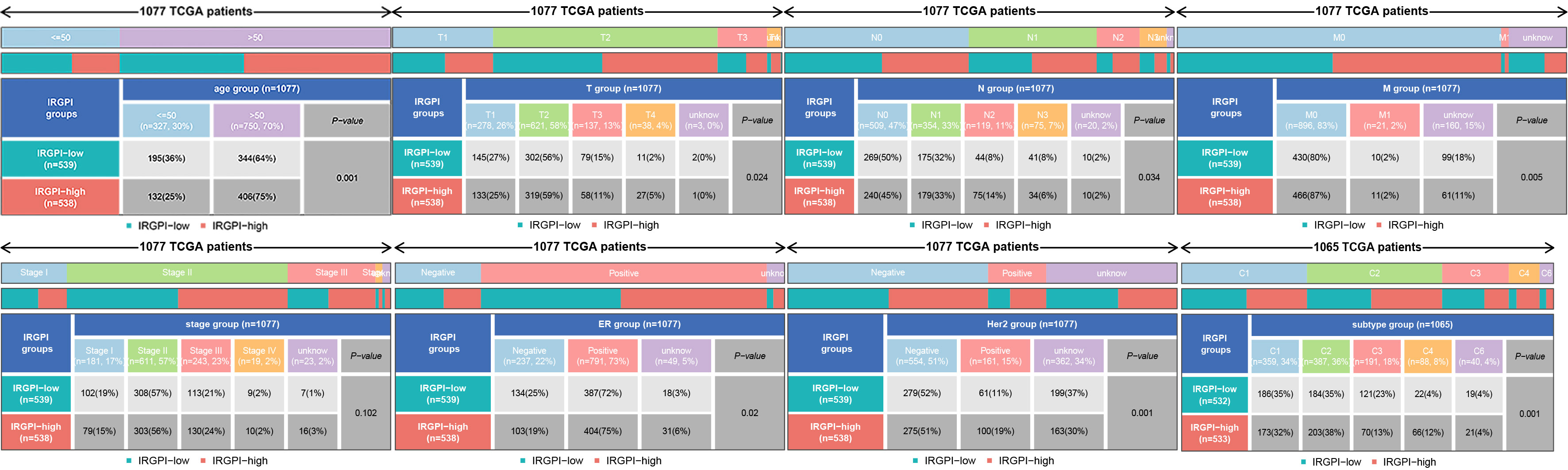

Supplement: Supplementary file 1 [file DataSheet_1.zip › Data Sheet 1 (1)/Supplementary/Supplementary Figure 5.jpg]

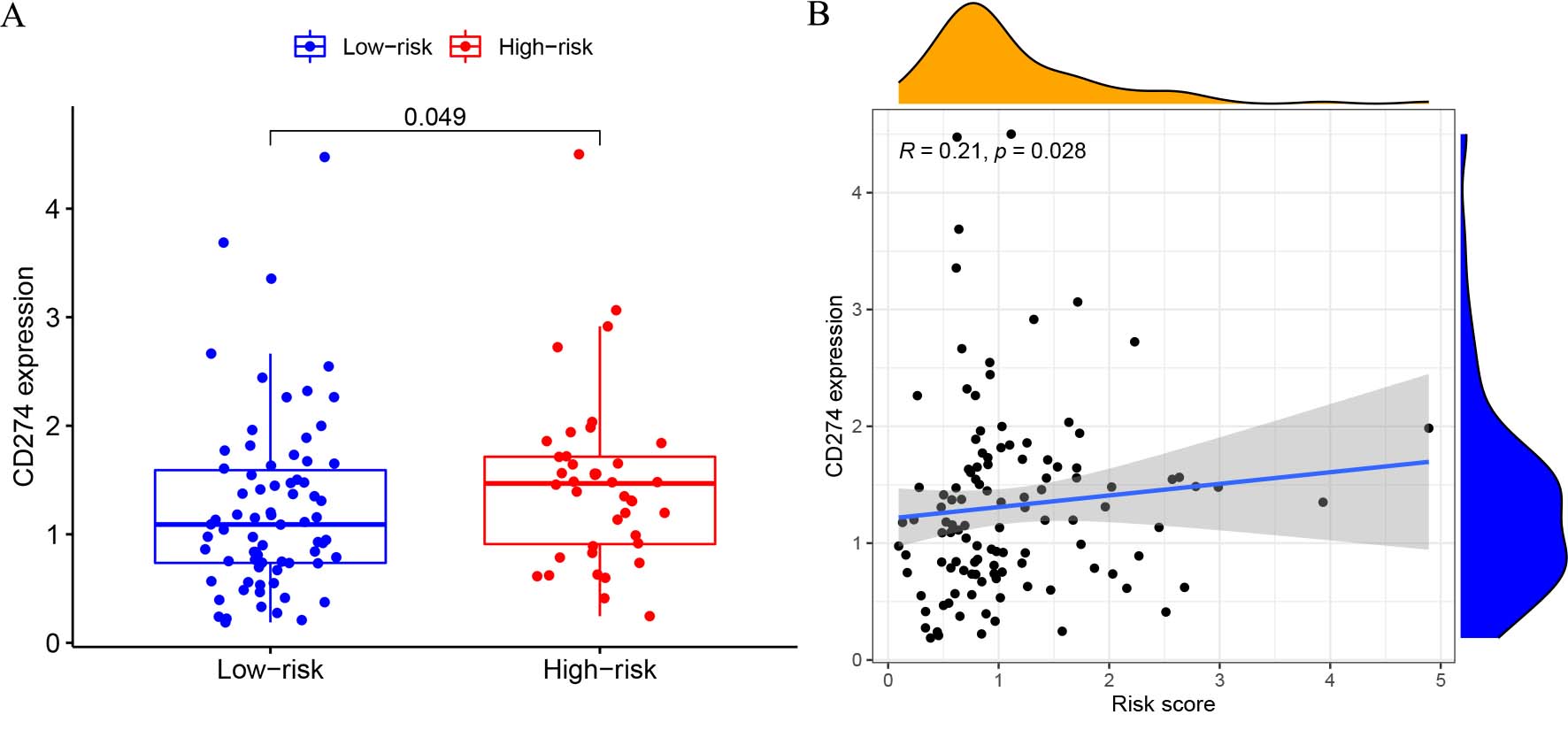

Supplement: Supplementary file 1 [file DataSheet_1.zip › Data Sheet 1 (1)/Supplementary/Supplementary Figure 6.jpg]
